# Biomarkers of equine asthma: A review of the current literature

**DOI:** 10.1007/s11259-026-11353-7

**Published:** 2026-06-24

**Authors:** Dorota Długopolska, Natalia Siwińska, Agnieszka Noszczyk-Nowak, Artur Niedźwiedź, Agnieszka Żak-Bochenek

**Affiliations:** 1 Department of Internal Medicine and Clinic of Diseases of Horses, Dogs and Cats, Faculty of Veterinary Medicine, Wrocław University of Environmental and Life Sciences, Grunwaldzki sq 47, Wroclaw, 50-366 Poland; 2 Department of Immunology, Pathophysiology and Veterinary Preventive Medicine, Faculty of Veterinary Medicine, Wrocław University of Environmental and Life Sciences, Grunwaldzki sq 47, Wroclaw, 50-366 Poland

**Keywords:** Equine Asthma, Biomarkers, Bronchoalveolar Lavage Fluid, Blood Samples, Respiratory Disease

## Abstract

Equine Asthma (EA) is a common, lower airway disease. Yet current diagnostic standards rely on bronchoalveolar lavage fluid (BALF) cytology, which is moderately invasive and not always feasible in field conditions. This review aimed to synthesize current evidence on candidate biomarkers of EA, with a primary focus on blood-based matrices and BALF, while also including data on biomarkers measured in tracheal wash (TW), saliva and exhaled breath condensate (EBC), where available. Searches were performed in PubMed, Scopus and Google Scholar, and original studies in horses with mild-to-moderate or severe EA reporting potential biomarkers were included. Across 57 unique studies, a broad spectrum of candidate biomarkers was evaluated. Blood-derived biomarkers, including surfactant protein D (SP-D), secretoglobin (SCGB), haptoglobin showed variable but generally limited specificity when assessed individually, although several combinations improved diagnostic accuracy. BALF-derived biomarkers more consistently reflected local airway inflammation and disease severity. Among these, neutrophil extracellular traps (NETs) related markers, neutrophil gelatinase-associated lipocalin (NGAL) and allergen-specific IgE profiles emerged as the most promising candidates, particularly in multimarker panels and phenotype-stratified analyses. Overall, current evidence supports the potential of integrated biomarker panels, especially those derived from BALF and complemented by blood-based markers, to refine the diagnosis and monitoring of EA, but robust validation in large, well-characterised study group is still required before routine clinical implementation.

## Introduction

Equine Asthma (EA) is a chronic lower respiratory tract disease classified into two phenotypes: mild to moderate Equine Asthma (mEA) and severe Equine Asthma (sEA). sEA occurs in 10–20% of the adult horse population (typically older than 7 years) in the Northern hemisphere (Hotchkiss et al. 2006, 2007). sEA is characterised by chronic cough, increased respiratory effort at rest, and exercise intolerance (Couëtil et al. 2016). The prevalence of mEA is estimated at 68–80% of the studied sport horse populations (Gerber et al. 2003; Allen et al. 2006; Ivester et al. 2018). mEA can affect horses of any age and usually presents with poor performance and intermittent coughing, without increased respiratory effort at rest (Couëtil et al. 2016). A direct progression from mEA to sEA has not been clearly established, and these forms should not be interpreted as consecutive disease stages. Although horses exhibiting occasional coughing and nasal discharge may have an increased risk of developing sEA, the mechanisms underlying the development and progression of EA are not fully understood (Bosshard and Gerber 2014; Couëtil et al. 2016). Many other aspects of EA also remain unclear. A similar challenge has persisted in human asthma, despite more research. Studies on human asthma may inform understanding of EA, but findings cannot be applied directly, as there are interspecies differences. Historically, human asthma was classified as either allergic or non-allergic. However, as understanding of the disease evolved, this classification was recognised as an oversimplification. Consequently, the T2-high and T2-low endotypes were introduced. Specifically, the T2-high endotype is associated with activation of allergen-specific Th2 cells, which drives the production of cytokines such as IL-4, IL-5, and IL-13. This endotype is characterised by eosinophilic and mast cell infiltration and increased mucosal production. Notably, within the T2-high endotype, four phenotypic subgroups have been identified, differing in disease severity (mild to very severe) and age of onset (early- to late-onset asthma). By contrast, the T2-low endotype is defined by the absence of type 2-driven inflammation, predominantly neutrophilic airway infiltrates, and limited responsiveness to corticosteroid treatment. This endotype is also commonly linked to obesity, smoking, and ageing (Hammad and Lambrecht 2021). The pathophysiology of EA is multifactorial and influenced by genetic predisposition, environmental exposures, infectious agents, and diverse immune regulatory pathways. EA has been reported across various breeds and horse types, regardless of use (Richard et al. 2014; Couëtil et al. 2016; Hansen et al. 2020). In addition to genetic factors, exposure to respirable organic dust, endotoxins, moulds, fungi, and ultrafine particles originating from hay, bedding, and stable air are paramount in inducing both mEA and sEA (Couëtil et al. 2016; Pirie 2017, 2018). When predisposed animals are exposed to airborne particles, their immune system responds in different ways involving Th1-, Th2-, and Th17-associated cytokine patterns. Investigations of immune pathways in sEA report variable, sometimes contradictory findings, underscoring the heterogeneity and complexity of the immune response. Some studies in sEA-affected horses show increased expression of IL-4 and IL-5 in bronchoalveolar lavage fluid (BALF) lymphocytes, which may indicate a Th2-type immune response (Lavoie et al. 2001). However, this profile is associated with airway neutrophilia rather than eosinophilia, distinguishing sEA from the typical presentation of human asthma (Lavoie et al. 2001; Cordeau et al. 2004). Other studies have reported increased expression of IL-1β, IL-8, interferon gamma (IFN-γ), tumor necrosis factor alpha (TNF-α) and IL-17 in BALF, suggesting the involvement of Th1 and/or Th17-mediated immune responses (Ainsworth et al. 2003, 2006). Mixed Th1/Th2 (Giguère et al. 2002; Horohov et al. 2005) or Th2/Th17 (Pacholewska et al. 2017, 2020; Lo Feudo et al. 2024) immune profiles have also been observed. Similar variability has been reported in studies on mEA. In horses affected by this phenotype, both symptomatic and asymptomatic, BALF analyses have shown increased expression of TNF-α, IL-1, and IFN-γ genes and higher TNF-α and IFN-γ protein concentrations, suggesting activation of a Th1-type immune response (Hughes et al. 2011; Richard et al. 2014; Hansen et al. 2020). However, subsequent studies indicated a Th2-type response, with increased IL-4 and IL-5 expression in the mastocytic form of mEA (Lavoie et al. 2011; Beekman et al. 2012). Recent flow-cytometric evidence further supports the view that sEA is characterised by a local type 3 immune response with a Th17-associated bias, and mEA may constitute a separate endotype with partially distinct pathogenic mechanisms (Gressler et al. 2022). Based on currently available evidence, different inflammatory pathways may reflect distinct endotypes, even when they lead to similar clinical phenotypes, complicating the understanding of EA pathophysiology. Therefore, consideration of endotypes is crucial for a more accurate understanding of this disease.

**Fig. 1:**
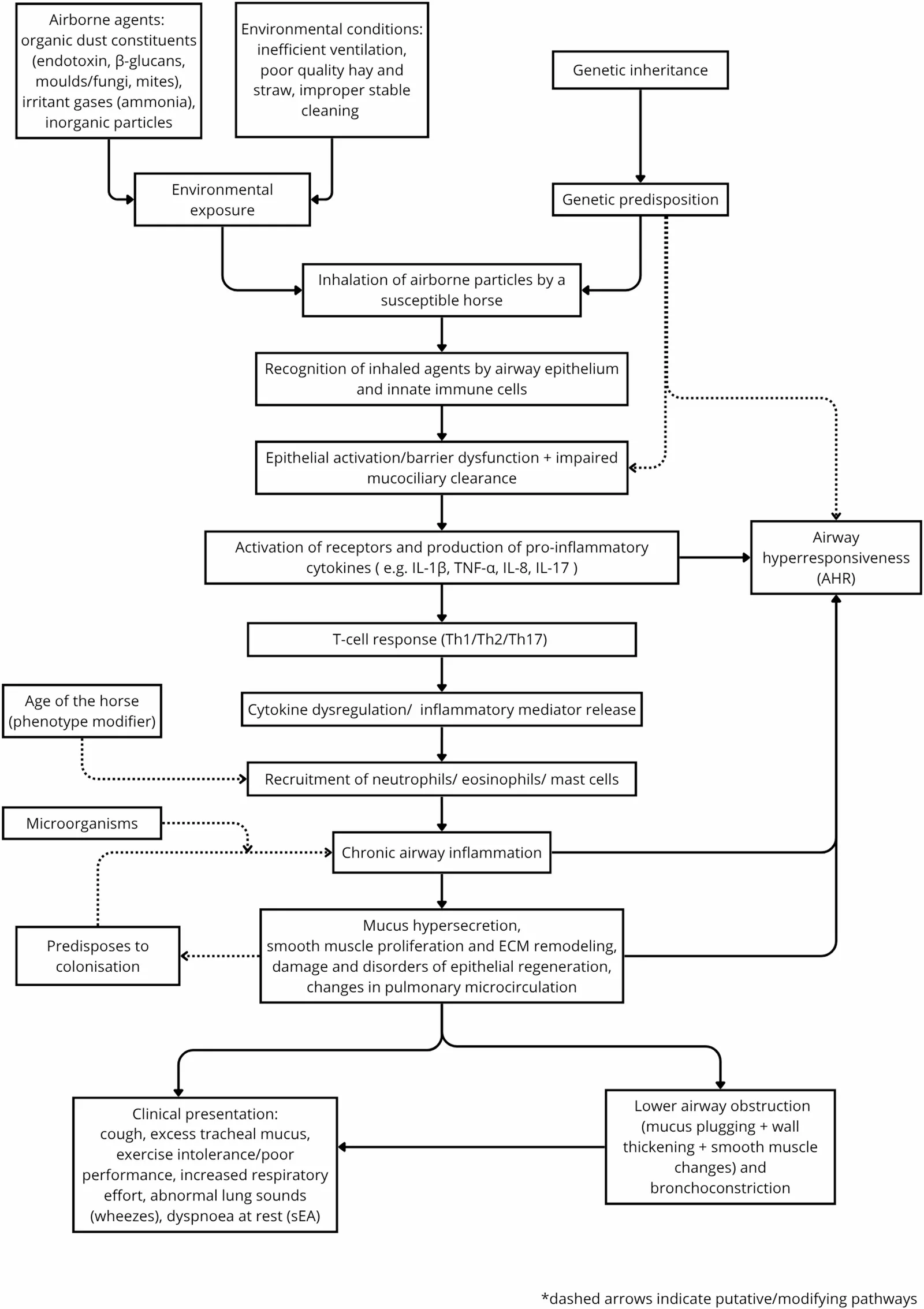
Simplified schematic overview of the pathophysiology of EA (Couëtil et al. 2016; Felippe 2016; Pirie 2017, 2018)

The airway epithelium also plays an important role in the pathological processes of EA. In affected horses, it undergoes marked remodelling, including thickening, hyperplasia, and goblet cell metaplasia, which increases mucus production. Club cells, important for epithelial regeneration, decrease in number. In severe inflammation, damage to ciliated cells can promote mucus retention and hinder the clearance of inhaled particles. The epithelium also actively participates in airway inflammation by producing chemokines and cytokines, such as IL-8, which recruits neutrophils to the airways (Tessier et al. 2017; Morini et al. 2021). A simplified overview of EA pathophysiology is presented in Fig. 1.

In humans, asthma diagnosis relies on clinical evaluation and pulmonary function testing, with spirometry being the gold standard. Additional assessments include measuring fractional exhaled nitric oxide (FeNO) (Bateman et al. 2008; Loewenthal and Menzies-Gow 2022). In horses, performing spirometry is challenging because it requires a suitable mask, which may not be well tolerated in some cases. The practical application of this procedure under field conditions remains challenging due to a lack of standardised protocols and limited access to measurement devices or dedicated data analysis systems (Burnheim et al. 2016; Kozłowska et al. 2022). Obtaining a full inspiratory and expiratory manoeuvre poses another difficulty (Lendl and Barton 2024). Furthermore, measurements collected during spontaneous breathing do not show significant differences between healthy horses and those with mEA (Couëtil et al. 2001). FeNO mainly reflects eosinophilic airway inflammation, whereas in horses with EA, neutrophilic and mastocytic inflammation are more frequently observed (Lavoie et al. 2011; Ivester et al. 2014; Davis and Sheats 2019). Because of interspecies differences in pathophysiology and clinical expression between human asthma and EA, diagnostic methods need to be tailored to each species. According to the American College of Veterinary Internal Medicine (ACVIM) consensus statement, EA diagnosis relies on clinical history, endoscopic assessment of tracheobronchial mucus, and cytological analysis of BALF. Based on BALF cytology, mEA may be further classified into neutrophilic, eosinophilic, mastocytic, or mixed cellular phenotypes, reflecting mild to moderate increases in these inflammatory cell populations. sEA is characterised by marked neutrophilia (> 25%) (Couëtil et al. 2016). Cytological criteria are presented in Table 1. BALF collection is a moderately invasive, sedation requiring test. The procedure can be performed using either an endoscope or a balloon catheter. A sterile saline solution is instilled into the bronchi in a volume appropriate to the horse’s size (usually between 250 and 500 ml) and then aspirated (Couëtil et al. 2016; Couëtil and Thompson 2020). A major limitation of this procedure is uncontrolled dilution, which may substantially influence both cytological results and measured biomarker concentrations (Couëtil et al. 2020). A recovery rate of at least 50% of the instilled fluid is generally regarded as necessary for BALF to be diagnostically useful. Poor recovery may result from severe bronchoconstriction in sEA or from technical factors. Other limitations include pre-analytical artefacts associated with delayed or improper transport, storage, or contamination. Some equine studies have utilised phosphate-buffered saline (PBS) during BALF collection or in later sample processing. This enhances cell preservation by providing a more suitable environment for recovered cells (Hansen et al. 2014; Barton et al. 2016a). BALF collection can also induce short-lived post-procedure effects, such as coughing, pressure-related mucosal injury, bleeding, transient neutrophilia lasting up to 48 h and occasional self-limiting fever within about 24 h (Couëtil et al. 2016; Pirie 2017, 2018; Couëtil and Thompson 2020). All these factors may complicate interpretation. In healthy horses, no clinically relevant residual airway inflammation was detected 72 h after BALF collection (Woodrow et al. 2024).

**Table 1:** BALF cytology criteria according to American College of Veterinary Internal Medicine (ACVIM) consensus (Couëtil et al. 2016)

Group	Cytological criteria (250 ml BALF infusion volume)
Healthy horses	Neutrophils ≤ 5% Eosinophils ≤ 1% Mast cells ≤ 2%
mEA	One or more of: Neutrophils > 10% Mast cells > 5% Eosinophils > 5%
sEA	Neutrophils > 25%

Because asthma is heterogeneous, standard diagnostic methods do not always fully reflect its complexity. Therefore, the search for biomarkers in both human asthma and EA is important for improving diagnostic precision and therapeutic strategies. The European Medicines Agency (EMA) defines a biomarker as an objective, quantifiable measure of a physiological process, a pathological process, or response to a treatment (European Medicines Agency (EMA) 2026). An ideal biomarker of EA should differentiate diseased from healthy individuals with high accuracy, reflect disease severity and treatment response, remain stable and reproducible over time, be easy to collect, and be cost-effective. Biomarkers may originate from various biological sources, each with specific advantages and limitations. Minimally invasive or noninvasive biomarkers are preferred, though they often face challenges with dilution, sensitivity, and lack of validated reference ranges (Fitzpatrick 2015). Nevertheless, given the complexity and diversity of the asthma pathophysiological mechanisms involved, it is unlikely that any single biomarker can represent all aspects of asthma across all patients.

Biomarkers can help classify patients by asthma phenotype and endotype (Narendra et al. 2019). In human asthma, type 2 inflammatory markers help identify T2-high asthma. These include blood and sputum eosinophils, FeNO, and serum periostin (Robinson et al. 2017). Endotype identification is key for modern targeted therapies. For instance, high eosinophil counts are associated with a favourable response to anti-IL-5 treatment (Hargreave and Nair 2011). Periostin concentrations have been investigated as predictors of response to anti-IL-13 therapy (Corren et al. 2011). Some biomarkers also have prognostic value by identifying patients at risk of severe disease or frequent exacerbations. For example, blood eosinophil count has been connected to exacerbation risk and poor asthma control. Changes in biomarker levels may reflect a biological response to treatment, showing their potential pharmacodynamic value (Narendra et al. 2019). Some markers, such as FeNO, also help monitor treatment adherence (Loewenthal and Menzies-Gow 2022). However, these examples come from human medicine and cannot be directly extrapolated to EA.

The presence and quantity of inflammatory cells (neutrophils, eosinophils or mast cells) in BALF are the current gold standard for diagnosing EA, but they are not optimal biomarkers. These cells provide information on airway inflammation but lack disease specificity (Winder and Von Fellenberg 1990; Newton and Wood 2002; Anderson and Singh 2018). Similar cytological patterns may occur in other respiratory diseases, particularly infectious conditions, but may also arise from different inflammatory pathways within EA itself. Interpretation may also be complicated if the horse has received prior pharmacotherapy, as certain medications (e.g., corticosteroids) may alter or mask cytological results. Additionally, secondary bacterial infection can modify the inflammatory profile and further hinder differentiation between infectious and noninfectious airway inflammation (Couëtil and Thompson 2020; Simões and Tilley 2023). Further challenges may arise when interpreting results that fall within the “grey zone” (5–10% neutrophils) as well as in cases of discrepancy between clinical signs and cytological findings (Couëtil et al. 2016). Given these limitations, there is growing interest in identifying biomarkers to improve the accuracy of diagnosing and monitoring EA. Accordingly, numerous studies have already been conducted and published investigating candidate biomarkers across different biological matrices and methodological approaches, reflecting the clinical relevance of EA and the need for more robust diagnostic tools. This expanding and heterogeneous evidence base provides the rationale for the present synthesis.

This review summarises current evidence on candidate biomarkers of EA, with particular emphasis on those investigated in blood and BALF. It synthesises studies evaluating a broad range of measurable biological components as potential tools to improve the diagnosis and characterisation of EA.

## Materials and methods

The literature search and reporting were informed by the PRISMA 2020 recommendations, where applicable to narrative syntheses. This work should be considered a narrative review with a systematic literature search rather than a full systematic review with meta-analysis. The primary aim was to summarise studies evaluating candidate biomarkers of EA measured in blood-based matrices (serum, plasma or whole blood) and BALF. Because most available studies assessed these two sample types, data extraction and synthesis were centred on blood-based matrices and BALF. Biomarkers measured in other matrices, such as tracheal wash (TW), saliva, and exhaled breath condensate (EBC), were included when available but treated as supplementary evidence and not tabulated separately due to their limited number and heterogeneity. Only original research articles involving horses were considered. Both experimentally induced and naturally occurring forms of EA were eligible, provided that biomarker measurements were reported.

In accordance with the ACVIM consensus statement (Couëtil et al. 2016) and recent literature (Pirie 2017, 2018), historical terms were aligned with current EA nomenclature: chronic obstructive pulmonary disease (COPD), recurrent airway obstruction (RAO), and heaves were classified as sEA, whereas inflammatory airway disease (IAD) was classified as mEA. This approach was applied consistently throughout data extraction, Tables 2 and 3, and the Discussion.

**Table 2:** Potential blood biomarkers of Equine Asthma (mEA/sEA), their direction of change (↑ increase; ↓ decrease; ↔ no change) and associated references

Biomarkers	Study	Nr of horses included in study	The asthma phenotype associated with the observed changes	Level changes	Main limitation	Conclusions
Cyclooxygenase products	(Gray et al. 1989)	5 horses in control group; 5 horses with sEA	sEA	Thromboxane B₂ (TXB₂) ↑ 6-keto-prostaglandin F1 alpha (6-keto-PGF_1α_) ↔ 9α, 11β-PGF_2_ ↔	Artefactual formation during collection may have affected measured TXB₂ levels.	Only plasma TXB₂ increased during sEA exacerbation, which may reflect inflammatory activation during sEA exacerbation. The other cyclooxygenase metabolites remained unchanged. Its clinical diagnostic value remains unconfirmed, and it currently has no established role in the diagnosis or phenotypic classification of EA.
Endothelin 1 (ET-1)	(Costa et al. 2009)	6 horses in control group; 6 with sEA	sEA	↑	High inter-individual variability in ET-1 concentrations and possible carry-over effects of prior bronchodilator or corticosteroid treatment.	ET-1 should be regarded primarily as a mediator involved in the pathogenesis of EA, particularly airway inflammation and bronchoconstriction, rather than as a validated diagnostic biomarker. Although its concentration may increase during sEA exacerbation, its clinical diagnostic value remains unconfirmed, and it currently has no established role in the diagnosis or phenotypic classification of EA.
8-Hydroxy-2-deoxyguanosine (8-OHdG)	(Niedzwiedz et al. 2015)	7 horses in control group; 7 horses with sEA	sEA	↑	The study was limited by short-term exposure and a single post-exposure measurement.	8-OHdG is a potential marker of oxidative DNA damage in sEA, but it is not disease-specific. Its clinical diagnostic value remains unconfirmed, and it currently has no established role in the diagnosis or phenotypic classification of EA.
Haptoglobin	(Lavoie-Lamoureux et al. 2012)	6 horses in control group; 6 horses with sEA	sEA	↑	The age of the horses might have influenced the study’s results.	Haptoglobin may serve as a potential marker for distinguishing sEA-affected horses from healthy controls. Elevated concentrations persisted even in asymptomatic horses, suggesting ongoing systemic inflammation during clinical remission.
	(Bullone et al. 2015)	10 horses in control group, 12 horses with mEA	mEA	↑	Haptoglobin may be elevated due to Exercise-Induced Pulmonary Haemorrhage (EIPH), requiring confirmation. On its own, it lacks sufficient specificity.	Haptoglobin may help differentiate mEA-affected horses from healthy controls. In combination SP-D differentiated mEA from controls with 100% sensitivity and 100% specificity at a log-transformed cut-off of 7.70 Its values in horses with mEA were higher than those reported previously for horses with sEA, which could support the hypothesis of disease progression from mEA to sEA.
	(Gy et al. 2019)	9 horses in control group, 14 with neutrophilic mEA, 10 with other pathologic conditions	neutrophilic mEA	↔	The study design did not allow evaluation of EIPH as a potential confounder in asthma diagnosis. Individually, the biomarkers did not show statistically significant differences between groups.	Diagnostic accuracy varied across marker sets. Haptoglobin > 6347 ng/mL reached a sensitivity of 79% and specificity of 41% (AUC = 0.6; 95% CI: 0.40–0.81). The combination of SP-D (> 58.9 ng/mL), haptoglobin (> 6347 ng/mL), and SCGB (< 25.7 ng/mL) achieved 100% specificity but moderate sensitivity (57%). Incorporating the clinical criterion Horse Owner Assessed Respiratory Signs Index (HOARS) = 2 further improved diagnostic precision, maintaining 100% specificity across different marker combinations.
	(Leclere et al. 2015)	31 Standardbred racehorses with exercise intolerance: 21 with mEA; 10 without mEA	mEA	↔	Race-speed exercise before sampling may have confounded the assessment of haptoglobin concentrations.	Haptoglobin concentrations did not differ significantly between groups. It suggests limited diagnostic utility for distinguishing mEA from other causes of exercise intolerance.
	(Christmann et al. 2022)	30 horses in control groups; 12 with sEA; 8 with neutrophilic mEA; 10 with eosinophilic mEA	sEA	↑	Incomplete group matching beyond age. Small sample sizes within each group due to stringent selection criteria and limited sample availability across institutions. No correction for multiple comparisons (multiple testing). Low BALF neutrophilia, particularly in mildly asthmatic horses, because a 5% cutoff was used to distinguish healthy from asthmatic horses.	Haptoglobin, particularly in combination with SP-D, may be more informative in sEA than in mEA phenotypes.
			neutrophilic mEA	↔		
			eosinophilic mEA	↔		
Serum Amyloid A (SAA)	(Lavoie-Lamoureux et al. 2012)	6 horses in control group; 6 horses with sEA	sEA	↑	Assigning arbitrary values to samples with very high or very low concentrations reduced the statistical power of the analysis.	Serum SAA increased significantly only on day 7. This suggests limited usefulness as a stand-alone diagnostic marker of sEA, but potential value as an indicator of acute systemic inflammation.
	(Bullone et al. 2015)	10 horses in control group, 12 horses with mEA	mEA	↑	A limitation of the study was the restricted quantification range of the ELISA assay, as SAA concentrations in four mEA horses exceeded the assay range and were therefore assigned the upper linear value of 3.30 µg/mL for statistical analysis.	Serum SAA concentrations were significantly increased in mEA compared with controls and correlated positively with BALF neutrophil percentage. No cut-off, sensitivity, or specificity was established for SAA alone, whereas the combination of SAA and SP-D achieved 100% sensitivity and 90% specificity at a log-transformed cut-off of 0.80.
	(Leclere et al. 2015)	31 racehorses with exercise intolerance: 21 with mEA; 10 without mEA	mEA	↔	A limitation of the study was the small sample size, which resulted in low statistical power for SAA (8%) and may have prevented the detection of a clinically relevant difference between groups.	SAA concentrations did not differ significantly between exercise-intolerant horses with mEA and those without lower airway inflammation. It suggests limited diagnostic utility for distinguishing mEA from other causes of exercise intolerance.
	(Lee et al. 2022)	6 horses in control group, 6 horses with sEA	sEA	↑	A limitation of the study was the small sample size, which may have reduced the ability to detect significant differences in serum SAA between asthmatic and non-asthmatic horses despite marked post-exposure increases in some individuals.	SAA concentrations increased after dust exposure, indicating systemic inflammation, but post-exposure values did not differ significantly between asthmatic and non-asthmatic horses. This suggests the limited value of SAA as a specific biomarker of sEA in this setting.
Surfactant protein D (SP-D)	(Richard et al. 2012)	20 horses in control group; 22 with mEA	mEA	↑	A limitation of the study was the use of a commercial ELISA kit developed for humans to measure equine SP-D, together with assessment of SP-D in serum only, without evaluation of the BALF/serum ratio.	SP-D concentrations were significantly higher in horses with mEA than in controls, both at rest and 60 min after exercise, whereas exercise itself had no significant effect on SP-D levels. A cut-off value of 48.0 ng/mL differentiated affected horses from controls with 82% sensitivity, 90% specificity, and an AUC of 0.923.
	(Bullone et al. 2015)	10 horses in control group, 12 horses with mEA	mEA	↑	A key limitation was that the effect of other causes of poor performance on serum SP-D concentrations remains unknown. This includes both respiratory and non-respiratory diseases.	SP-D concentrations were significantly increased in mEA compared with controls and outperformed the other serum biomarkers evaluated. In combination with haptoglobin, SP-D differentiated mEA from controls with 100% sensitivity and 100% specificity at a log-transformed cut-off of 7.70.
	(Gy et al. 2019)	9 horses in control group, 14 with neutrophilic mEA, 10 with other pathologic conditions	neutrophilic mEA	↑	A limitation of the study was the narrow diversity of asthma phenotypes, as the analysis focused on neutrophilic asthma, preventing assessment of SP-D in mastocytic or eosinophilic subtypes.	SP-D concentrations were significantly increased in horses with neutrophilic mEA. This was especially true in those with ≥ 15% neutrophils in BALF. Using the corrected cut-off of > 58.9 ng/mL, SP-D alone differentiated neutrophilic mEA from healthy and pathologic controls with 57% sensitivity and 84% specificity (AUC 0.75). Combining SP-D with SCGB increased specificity to 95%, but sensitivity decreased to 50%.
	(Christmann et al. 2022)	30 horses in control groups; 12 with sEA; 8 with neutrophilic mEA; 10 with eosinophilic mEA	sEA	↑	Incomplete group matching beyond age. Small sample sizes within each group due to stringent selection criteria and limited sample availability across institutions. No correction for multiple comparisons (multiple testing). Low BALF neutrophilia, particularly in mildly asthmatic horses, because a 5% cutoff was used to distinguish healthy from asthmatic horses.	Serum SP-D concentrations were significantly increased only in horses with sEA, whereas no significant increase was detected in mEA. Using the previously proposed cut-off of > 43 ng/mL, 10 of 12 horses with sEA exceeded this threshold, suggesting that serum SP-D is more informative in sEA than in mEA.
			neutrophilic mEA	↔		
			eosinophilic mEA	↔		
Secretoglobin (SCGB)	(Gy et al. 2019)	9 horses in control group, 14 with neutrophilic mEA, 10 with other pathologic conditions	mEA (neutrophilic form)	↓	A limitation of the study was the narrow diversity of asthma phenotypes, as the analysis focused on neutrophilic asthma, preventing assessment of SCGB in mastocytic or eosinophilic subtypes.	SCGB concentrations were lower in horses with neutrophilic mEA. A cut-off of less than 25.7 ng/mL differentiated affected horses from healthy and pathologic controls, with 77% sensitivity and 47% specificity. When combined with SP-D, specificity increased to 95%, but sensitivity dropped to 50%. This suggests SCGB has limited value as a stand-alone biomarker but may be useful in multimarker panels.
C-reactive protein (CRP)	(Leclere et al. 2015)	31 racehorses with exercise intolerance: 21 with mEA; 10 without mEA	mEA	↔	A limitation of the study was the small sample size, which resulted in low statistical power for CRP (31%) and may have prevented the detection of a clinically relevant difference between groups.	CRP concentrations did not differ significantly between exercise-intolerant horses with mEA and those without lower airway inflammation. Current support for its role as a candidate EA biomarker remains limited.
Leucine‑rich repeats and calponin‑homology domain‑containing 4 (Lrch4)	(Mainguy-Seers et al. 2022)	8 horses with sEA	sEA	↑ (expression)	There was no control group of healthy horses in the study.	MAST4 and LRCH4 were significantly upregulated in neutrophil-derived extracellular vesicles (EV) during sEA exacerbation. This suggests a potential association with disease activity. Their diagnostic value remains unconfirmed.
Microtubule‑associated serine/threonine‑protein kinase 4 (MAST4)						
Soluble triggering receptors expressed on myeloid cells-1 (sTREAM-1)	(Bullone et al. 2015)	10 horses in control group, 12 horses with mEA	mEA	↔	A limitation was that the study focused on relatively mild airway inflammation, which may have reduced the likelihood of detecting changes in serum sTREM-1.	Serum sTREM-1 concentrations did not differ significantly between mEA and control horses. This suggests that sTREM-1 does not currently appear to have relevant biomarker potential in this setting.
Cytokines	(Lavoie-Lamoureux et al. 2012)	6 horses in control group; 6 horses with sEA	sEA	IL-10, CCL2, IFN-γ ↔ IFN-α, IL-2 below detection	The study was limited by a small sample size.	IL-10, CCL2, and IFN-γ showed no significant between-group differences. IFN-α and IL-2 remained undetectable. Current support for their role as candidate EA biomarkers remains limited.
	(Hamouzová et al. 2023)	14 horses with mEA; 9 horses with sEA	sEA	IL-10, IL-4, IL-17 IFN-γ ↔	The study was limited by a small sample size, lack of a healthy control group, and age-related confounding.	IFN-γ, IL-4, IL-10 and IL-17 did not differ significantly between mEA and sEA, suggesting limited value as biomarkers for differentiating clinical severity.
			mEA			
MicroRNA (miRNA)	(Pacholewska et al. 2017)	34 horses in control group; 37 horses with sEA	sEA	Altered (↓miR-128, ↓miR-744)	The study’s limitations include the small log-fold changes in the differentially expressed microRNAs, challenging their use as specific diagnostic biomarkers, alongside confounding effects from sample hemolysis.	Eleven serum miRNAs were differentially expressed in sEA. Downregulated serum miR-128 and miR-744 may be more relevant to disease-related immune pathways than to clinical diagnosis. Their diagnostic value remains unconfirmed.
Procalcitonin (PCT)	(Barton et al. 2016a)	15 horses in control group; 21 with sEA; 16 with mEA; 12 with chronic interstitial pneumopathy (CIP) - in whole study but PCT were measured only in 17 horses − 10 controls and 7 with respiratory diseases	mEA/sEA	↑	Measurements were limited to only 17 horses. This small sample size is insufficient to fully assess its usefulness as a biomarker or to draw statistically reliable conclusions.	Plasma PCT was increased in horses with chronic respiratory disease (sEA, mEA, CIP), and appeared more informative than BALF PCT, but its diagnostic value remains unconfirmed.
Vitamin D (25(OH)D)	(Mainguy-Seers et al. 2024)	15 horses in control group; 14 horses with mEA; 16 with sEA	mEA/sEA	↔	The study was limited by its retrospective design, incomplete control of confounding factors affecting vitamin D status, lack of a priori power calculation, and possible misclassification of some control horses with airway neutrophilia.	Serum 25(OH)D concentrations did not differ significantly between mEA, sEA and control horses. It suggests that vitamin D status does not currently play a major role in distinguishing EA from other conditions and has no evident diagnostic value as a serum biomarker.
Myostatin (MSTN)	(Kowalik et al. 2024)	10 healthy recreational horses; 12 healthy racehorses; 15 horses with sEA	sEA	↔	The authors concluded that factors such as breed, age, and physical activity likely influence plasma MSTN concentrations more than the presence of EA.	MSTN concentrations did not differ significantly between horses with sEA and age- and use-matched healthy adult leisure horses, although they were higher than in young healthy Thoroughbred racehorses. Current support for its role as a candidate EA biomarker remains limited.
Redox biomarkers	(Hansen et al. 2025)	37 horses in control group; 26 with mastocytic mEA; 29 with neutrophilic mEA; 25 with sEA	mEA/sEA	Glutathione reductase (GSHred), Advanced Oxidation Protein Products (AOPP), Superoxide dismutase (SOD) ↔	A limitation of the study was that the serum findings were derived from a cross-sectional comparison without diagnostic performance analysis, and several redox markers showed no clear group separation despite biological plausibility.	Serum GSHred, AOPP, and SOD concentrations did not differ significantly among the EA groups. Their clinical diagnostic value remains unconfirmed, and they currently have no established role in the diagnosis or phenotypic classification of EA.
			mastocytic mEA	Trolox Equivalent Antioxidant Capacity (TEAC), Ferric Reducing Antioxidant Power (FRAP) ↑		Horses with mastocytic mEA had significantly higher serum TEAC concentrations compared to healthy, neutrophilic mEA, and sEA groups. FRAP concentrations were significantly higher in the mastocytic mEA group compared with the neutrophilic mEA group. Its clinical diagnostic value remains unconfirmed, and it currently has no established role in the diagnosis or phenotypic classification of EA.
Neutrophil gelatinase-associated lipocalin (NGAL)	(Hansen et al. 2024)	27 horses in control group; 11 horses with neutrophilic mEA; 13 horses with mastocytic mEA; 4 mixed granulocytic with mEA, 11 with sEA	mEA/sEA	↔	Serum NGAL concentrations and haematology results were available only for a subset of the study population, specifically 66 of the 227 (29%) horses analysed.	NGAL concentrations did not differ between groups and did not correlate with BALF NGAL or BALF neutrophils. Current support for its role as a candidate EA biomarker remains limited. Serum NGAL level reflects systemic rather than airway neutrophilic inflammation.
Neutrophil cellular cholesterol	(Meiseberg et al. 2025)	8 horses in control group; 7 with sEA; 11 with mEA (7 with moderate EA; 4 with mild EA)	mEA (moderate) /sEA	↓	The study was limited by the small sample size, especially in mild EA, and by analytical constraints in lipid profiling, particularly the limited accuracy of LDL measurements.	Plasma total cholesterol decreased with EA severity and was lowest in sEA. Its clinical diagnostic value remains unconfirmed, and it currently has no established role in the diagnosis or phenotypic classification of EA.
Allergen-specific antibodies	(Schmallenbach et al. 1998)	3 horses in control group; 16 horses with sEA	sEA	IgE and IgG antibodies specific for *Aspergillus fumigatus* ↔	A limitation of the study was the small sample size of the control group relative to the affected group.	Serum *Aspergillus fumigatus-*specific IgE and IgG were not significantly different between groups. Their levels did not correlate with BALF concentrations. Current support for their role as candidate EA biomarkers remains limited.
	(White et al. 2019)	58 horses in control group; 34 with sEA; 23 with sEA and Insect Bite Hypersensitivity (IBH); 23 with IBH	sEA	allergen-specific IgE profile (protein microarray) ↑	Serum IgE profiles were strongly influenced by environmental exposure. The model was panel-based rather than driven by a single stand-alone biomarker.	Serum allergen-specific IgE profiling combined with PLS-DA discriminated horses with sEA from controls, with the strongest contributors including latex proteins (Hev b 11, Hev b 6.02, Hev b 5.0101), rAsp f 8 and Hel as 7. Notably, diagnostic performance was high in environmentally matched horses (cross-validated sensitivity 0.94, specificity 1.00). In contrast, it remained lower in the larger environmentally mixed groups (sensitivity 0.735, specificity 0.865).
	(Wyler et al. 2023)	39 horses in control group; 44 horses with EA	mEA/sEA	allergen-specific IgE (protein microarrey; 9/23 analytes) ↑	A major limitation of serum allergen-specific IgE was that most significant analytes were higher in control horses than in asthmatic horses, which likely explains the poor diagnostic performance of the serological model. This suggests that serum IgE may reflect environmental allergen exposure rather than true asthma-related disease processes. The asthmatic horses in this study were not divided by asthma phenotype, so potential differences among subtypes could not be assessed.	Serum allergen-specific IgE showed limited and inconsistent diagnostic utility in horses with asthma. Although 9 of 23 allergen-specific IgEs included in the final serum model were increased in asthmatic horses, most significant analytes were not consistently elevated, and the overall predictive performance of the serum model was poor (AUC 0.585). These findings indicate that serum IgE profiles are markedly less informative than BALF profiles and should be interpreted with caution.
Circulating immune complexes (CICs)	(Slowikowska et al. 2021)	6 horses in control group, 6 untreated horses with sEA; 6 horses with glucocorticoid‑treated sEA	sEA	↔	The study was limited by a small sample size and the absence of significant between-group differences at individual time points.	CICs did not differ significantly between groups at individual time points. Current support for their role as candidate EA biomarkers remains limited. However, a significant decrease was observed in the treated group and a significant increase in the untreated group by day 30, suggesting potential value for monitoring.
Dexamethasone-induced protein (gen DEXI)	(Mason et al. 2018)	42 horses in control group; 40 with sEA	sEA	↔ (expression)	A limitation of the study was that DEXI expression was assessed only in PBMCs, which may not fully reflect gene regulation in more disease-relevant airway tissues.	Although several sEA-associated SNPs regulated DEXI expression in PBMCs under baseline conditions, DEXI expression itself was not associated with disease status. Current support for its role as a candidate EA biomarker remains limited.
Hyaluronic acid (HA)	(Höglund et al. 2024)	15 horses in control group; 10 horses with neutrophilic mEA; 5 with sEA	sEA	↔	The study was limited by grouping based on airway cytology rather than the full clinical picture, classification restricted to neutrophilic airway inflammation despite mixed inflammatory patterns in some horses, uncontrolled environmental aspects such as stabling and transport, possible misclassification of horses in remission as controls, a small number of endobronchial biopsy (EBB) samples, HA molecular size assessment performed only in pooled samples precluding statistical analysis.	HA concentrations were not statistically different between groups. Current support for its role as a candidate EA biomarker remains limited.
			neutrophilic mEA	↔		

**Table 3:** Potential BALF biomarkers of Equine Asthma (mEA/sEA), their direction of change (↑ increase; ↓ decrease; ↔ no change) and associated references

Biomarkers	Study	Nr of horses included in study	The asthma phenotype associated with the observed changes	Level changes	Main limitations	Conclusions
Arachidonate metabolites	(Watson et al. 1992)	7 horses in control group; 4 horses with sEA	sEA	Prostaglandin E2 (PGE_2_) ↑	The study was limited by a small sample size. Rapid leukotriene degradation in the bronchial lumen may also have obscured true differences in LTB₄ and LTC₄ concentrations.	PGE₂ was significantly increased in sEA, suggesting a possible association with airway inflammation, but its stand-alone diagnostic value remains unclear.
				Prostaglandin F (PGF) ↑		PGF was significantly increased in sEA, suggesting possible association with disease-related airway changes, although its diagnostic usefulness remains unclear.
				6-keto-PGF_1α_; thromboxane B2 (TxB_2_); leukotriene B4 (LTB_4_), prostaglandin D2 (PGD_2_); leukotriene C4 (LTC_4_) ↔		The remaining arachidonate metabolites did not show significant differences between groups. Current support for their role as candidate EA biomarkers remains limited.
Histamine	(Hare et al. 1999)	5 horses in control group; 5 horses with sEA	sEA	↑	A limitation of the study was that only a limited range of fungal and microbial allergens was tested. This may not fully represent the complex environmental exposure encountered by horses under natural conditions.	Histamine release from pulmonary mast cells was significantly greater in horses with sEA than in healthy controls in response to selected fungal allergens, particularly *Aspergillus fumigatus* and *Alternaria tenuis*, both during remission and exacerbation. This suggests that histamine release reflects allergen-induced mast cell hyperreactivity in sEA, but its clinical diagnostic value as a biomarker remains unconfirmed.
Secretoglobin (SCGB)	(Miskovic Feutz et al. 2015)	10 horses in control group; 19 horses with mEA; 22 with sEA;	sEA	↓	A limitation of the study was the difference in duration of antigen exposure between the experimental and naturally occurring sEA groups.	SCGB expression was significantly lower in horses with sEA than in healthy controls and horses with mEA, and was most markedly decreased in naturally occurring sEA. These findings suggest that secretoglobin reflects local airway disease severity and may help distinguish severe from milder forms of EA, although its clinical diagnostic performance remains to be established.
Transferrin (TF)	(Miskovic Feutz et al. 2015)	10 horses in control group; 22 with sEA; 19 with mEA	sEA	↓	A limitation was that BALF TF may originate from plasma exudation and local pulmonary production, making its source difficult to distinguish without parallel plasma and BALF measurements.	TF expression was lower in horses with sEA than in horses with mEA, and was similarly decreased in naturally occurring and experimentally induced sEA. This suggests an association with severe airway inflammation, although its clinical diagnostic value remains to be established.
Redox biomarkers	(Hansen et al. 2025)	37 horses in control group; 26 with mastocytic mEA; 29 neutrophilic mEA; 25 with sEA	mEA/sEA (neutrophilic form)	TEAC ↓	Several redox-related biomarkers could not be reliably measured in BAL fluid due to insufficient concentration or methodological challenges.	TEAC, SOD, and AOPP concentrations decreased with increasing EA severity, with the lowest values observed in sEA. These findings support an association between local redox imbalance and neutrophilic airway inflammation, although their clinical diagnostic value remains to be established.
				AOPP ↓		
				SOD ↓		
				GSHred ↔		GSHred and FRAP concentrations did not differ significantly among the EA groups. Current support for their role as candidate EA biomarkers remains limited.
				FRAP ↔		
Myristoylated Alanine-Rich C Kinase Substrate (MARCKS)	(Conley et al. 2025)	11 horses in control groups; 10 with mEA; 22 with sEA	mEA/sEA	↑	A limitation of the study was the variability in asthma exacerbation status between the two populations. This variability made direct comparisons difficult. It may also have affected the relationship between MARCKS levels and BALF neutrophils.	Normalised MARCKS protein levels were increased in BAL cell lysates from horses with EA, especially sEA, and MARCKS inhibition reduced zymosan-stimulated respiratory burst in alveolar macrophages and neutrophils. This suggests a role in neutrophilic airway inflammation, but its clinical diagnostic value remains unconfirmed.
Myeloperoxidase (MPO)	(Art et al. 2006)	6 horses in control group; 7 horses with sEA	sEA	↑	A limitation of the study was that remission was assessed only after 2 months on pasture, so it remained unclear whether BALF MPO concentrations would normalise with longer antigen avoidance or remain persistently elevated over time.	MPO concentrations were increased in horses with sEA, remained elevated during remission, and were associated with neutrophilic airway inflammation. This suggests that MPO may reflect persistent lower airway neutrophil activation, but its clinical diagnostic value remains unconfirmed.
Neutrophil gelatinase-associated lipocalin (NGAL)	(Hansen et al. 2024)	73 horses in control group; 36 horses with neutrophilic mEA; 47 with mastocytic mEA; 56 with sEA	mEA/sEA	↑	The study was limited by overlap in BALF NGAL concentrations between groups. It also had only moderate diagnostic validity for some comparisons. In addition, it could not distinguish between neutrophilic and mastocytic MEA.	NGAL concentrations were significantly higher in mEA than in control horses and in sEA than in mEA. No significant difference was found between neutrophilic and mastocytic mEA. Diagnostic accuracy was moderate for control versus mEA (AUC = 0.72) and higher for control versus sEA (AUC = 0.88), suggesting that BALF NGAL may help differentiate mEA and sEA.
Tumor necrosis factor alpha (TNF-α)	(Giguère et al. 2002; Riihimäki et al. 2008c; Hughes et al. 2011; Beekman et al. 2012; Richard et al. 2014; Montgomery et al. 2018; Woodrow et al. 2023)	Multiple studies with different methodologies	mEA/sEA	inconsistent	A key limitation is the marked heterogeneity between studies in matrix, disease stage, and analytical endpoint (mRNA, protein, or bioactivity), which prevents direct comparison and precludes a universal diagnostic cut-off. TNF-α is also modified by corticosteroid treatment, limiting its stability as a biomarker.	TNF-α reflects airway inflammatory activity in EA, but findings are inconsistent across studies, with reported increases, no change, or even decreases depending on disease phenotype, stage, matrix, and assay. Therefore, it currently has no established value as a stand-alone diagnostic biomarker.
Interferon-gamma (IFN-γ)	(Woodrow et al. 2023)	11 horses in control group; 5 with mEA, 14 with sEA	sEA	↑	Researchers emphasised that mEA and sEA likely comprise multiple endotypes and phenotypes, which complicates full delineation of their pathogenesis and the establishment of clinically meaningful disease categories.	IFN-γ concentrations were significantly higher in sEA than in healthy horses, but no significant difference was found between mEA and sEA. IFN-γ, therefore, appears to reflect the immunopathology of sEA rather than serve as a validated diagnostic biomarker. Its clinical diagnostic value remains unconfirmed.
	(Giguère et al. 2002)	4 horses in control group; 4 horses with sEA	sEA	↑	The study was limited by a small sample size. In addition, reduced allergen exposure during the post-treatment period may have confounded the effect of inhaled fluticasone on cytokine expression.	IFN-γ mRNA expression increased during sEA exacerbation in one of two trials. It was higher in sEA-affected horses than in controls during the post-treatment period. The IFN-γ/IL-4 ratio was significantly higher after treatment in treated horses than in untreated horses. This suggests potential usefulness as a marker of therapeutic response.
	(Lo Feudo et al. 2024)	1 horse in control group; 6 with neutrophilic EA; 8 mixed granulocytic EA	neutrophilic and mixed granulocytic form of EA	↔ (expression)	The study was limited by a small sample size and the inclusion of only one horse as a healthy reference for normalisation. The study focused on differences between asthma subtypes in affected horses rather than on direct comparisons with a representative healthy control group.	IFN-γ mRNA expression did not differ between neutrophilic and mixed granulocytic EA, was positively correlated with BALF mast cell percentage, and was not associated with lung function. Its clinical diagnostic value remains unconfirmed, and it currently has no established role in distinguishing EA subtypes.
IL-1β	(Lo Feudo et al. 2024)	1 horse in control group; 6 with neutrophilic EA; 8 mixed granulocytic EA	neutrophilic and mixed granulocytic form of EA	↔ (expression)	The study was limited by a small sample size and the inclusion of only one horse as a healthy reference for normalisation. The study focused on differences between asthma subtypes in affected horses rather than on direct comparisons with a representative healthy control group.	IL-1β mRNA expression did not differ between neutrophilic and mixed granulocytic EA, but was positively correlated with BALF neutrophil percentage and increased expiratory resistance. Its clinical diagnostic value remains unconfirmed, and it currently has no established role in distinguishing EA subtypes.
	(Davis and Sheats 2021)	5 horses in control group; 5 with neutrophilic mEA; 5 with mastocytic mEA	mastocytic and neutrophilic mEA	↑ (expression)	A key limitation was that IL-1β is strongly influenced by post-transcriptional and post-translational mechanisms. Therefore, increased protein levels may reflect enhanced protease-mediated activation in neutrophilic airways rather than increased IL-1β mRNA expression alone.	IL-1β mRNA was increased in both neutrophilic and mastocytic mEA compared with controls, whereas IL-1β protein was significantly higher only in neutrophilic mEA. This suggests that IL-1β may better reflect neutrophilic than mastocytic airway inflammation, although its clinical diagnostic value remains unconfirmed.
IL-4	(Lo Feudo et al. 2024)	1 horse in control group; 6 with neutrophilic EA; 8 mixed granulocytic EA	neutrophilic and mixed granulocytic form of EA	↑ (expression)	The study was limited by a small sample size and the inclusion of only one horse as a healthy reference for normalisation. The study focused on differences between asthma subtypes in affected horses rather than on direct comparisons with a representative healthy control group.	IL-4 mRNA expression was significantly higher in mixed granulocytic than in neutrophilic EA, was positively correlated with BALF mast cell percentage, and inversely correlated with reactance. This suggests a potential association with mastocytic EA, but its clinical diagnostic value remains unconfirmed.
CXCL-8/IL-8	(Franchini et al. 2000; Giguère et al. 2002; Ainsworth et al. 2003, 2006, 2009; Pietra et al. 2007; Riihimäki et al. 2008a; Padoan et al. 2013; Hansen et al. 2014; Woodrow et al. 2023)	Multiple studies with different methodologies	mEA/sEA	↑	A major limitation of IL-8 studies is the marked methodological heterogeneity, including differences in sample type, mRNA versus protein measurement, normalisation strategies, and timing of sampling relative to allergen exposure, which makes results difficult to compare across studies and prevents the establishment of a universal diagnostic cut-off. In addition, IL-8 appears less consistent in milder EA phenotypes, limiting its value as a stand-alone diagnostic marker.	IL-8 is one of the most consistently studied BALF biomarkers in EA and reflects neutrophilic airway inflammation. Its concentrations generally increase in parallel with BALF neutrophilia and disease severity, particularly in sEA. However, its value as a stand-alone diagnostic marker is limited. This limitation arises from the absence of a universal cut-off, methodological variability, inconsistent findings in milder phenotypes, and strong dependence on allergen exposure and sampling timing. Currently, IL-8 is better regarded as a marker of inflammatory activity and for disease monitoring, rather than as an independent diagnostic test for EA.
IL-10	(Beekman et al. 2012)	10 horses in control group; 8 with mastocytic mEA, 9 with neutrophilic mEA	mEA	↑ (expression)	A limitation of the study was that IL-10 was assessed only at the mRNA level in mixed BALF cell populations, without confirmation at the protein level, so post-transcriptional regulation and differences between specific cell types could not be evaluated.	IL-10 mRNA expression in BALF cells was increased in mEA overall, but not significantly in mastocytic or neutrophilic mEA analysed separately. Its clinical diagnostic value remains unconfirmed.
IL-17 A	(Lo Feudo et al. 2024)	1 horse in control group; 6 with neutrophilic EA; 8 mixed granulocytic EA	neutrophilic and mixed granulocytic form of EA	↔ (expression)	The study was limited by a small sample size and the inclusion of only one horse as a healthy reference for normalisation. The study focused on differences between asthma subtypes in affected horses rather than on direct comparisons with a representative healthy control group.	IL-17 A mRNA expression did not differ between neutrophilic and mixed granulocytic EA, but was inversely correlated with expiratory reactance. Its clinical diagnostic value remains unconfirmed, and it currently has no established role in distinguishing EA subtypes.
Neurokinin A (NKA)	(Morini et al. 2021)	5 horses in control group; 6 horses with sEA	sEA	↑ (expression)	NKA was assessed by semiquantitative immunohistochemistry on endobronchial biopsies, which limits precision and does not provide absolute quantification of mediator expression.	Bronchial epithelial NKA immunoreactivity was significantly stronger in horses with sEA than in controls during late exacerbation and remission. No significant differences were found during the asymptomatic or early exacerbation phases. These findings suggest that NKA is associated with bronchial mucosal changes in sEA. However, its clinical diagnostic value remains unconfirmed.
CXCL13	(Sage et al. 2024)	5 horses in control group; 6 horses with sEA	sEA	↑ (expression)	The study was limited by a small sample size and by the lack of validation of CXCL13 expression beyond the transcriptomic level, including no assessment of protein concentrations or diagnostic accuracy.	CXCL13 expression was upregulated in intermediate monocytes in sEA, suggesting involvement in the Th17-driven immunopathology of the disease. Its clinical diagnostic value remains unconfirmed.
KIT (c‑KIT/CD117)	(Davis and Sheats 2021)	5 horses in control group; 5 with neutrophilic mEA; 5 with mastocytic mEA	mastocytic and neutrophilic mEA	↑ (expression)	Although KIT mRNA was markedly increased, immunoblot analysis did not confirm significant differences at the protein level. This was likely due to KIT protein instability, including rapid degradation or internalisation, which may limit its current diagnostic applicability.	KIT mRNA expression was significantly increased in mastocytic mEA compared with the control group. This finding indicates an association with the mastocytic subtype. In contrast, only a moderate increase in neutrophilic mEA was observed. Its clinical diagnostic value remains unconfirmed.
Matrix metalloproteinases/tissue inhibitors of metalloproteinases (MMPs/TIMPs)	(Barton et al. 2015)	15 healthy horses; 17 with sEA; 18 with mEA; 11 with CIP	mEA/sEA	MMP-2, TIMP-1, TIMP-2, MMP-9, MMP-8 ↑	A major limitation was the definition of the study groups, as samples were collected from clinical patients, which made it difficult to clearly distinguish mEA from remission-phase sEA.	MMP-2, TIMP-1, and TIMP-2 were increased in sEA and mEA but not in CIP, whereas MMP-9 and MMP-8 were increased in all disease groups and were highest in sEA. MMP: TIMP ratios, particularly MMP-8:TIMP-1 and MMP-8:TIMP-2, appeared to better reflect the imbalance between matrix degradation and repair than individual analytes alone. Overall, these findings support their association with chronic airway inflammation and remodelling. Their clinical diagnostic value remains unconfirmed.
	(Hamouzová et al. 2023)	14 horses with mEA; 9 horses with sEA	sEA	MMP-9 ↑	The study was limited by a small sample size, lack of a healthy control group, and age-related confounding.	MMP-9 is significantly higher in sEA than in mEA. It is increased in neutrophilic/mixed versus mastocytic airway inflammation. MMP-9 shows a strong positive correlation with BALF neutrophil percentage and clinical score. These findings support MMP-9 as a severity- and inflammation-linked airway biomarker.
	(Davis and Sheats 2021)	5 horses in control group; 5 with neutrophilic mEA; 5 with mastocytic mEA	mastocytic and neutrophilic mEA	MMP-2 ↑	A key limitation was the biological nature of MMP-2 as a secreted and dynamically regulated protease, which makes cell lysate measurements alone insufficient for full interpretation.	MMP-2 differed between inflammatory subtypes of mEA. Its mRNA expression was increased in mastocytic mEA compared with the control group, whereas MMP-2 protein levels in BAL cell lysates were significantly higher in neutrophilic than in mastocytic mEA. This indicates biological relevance at the subtype level, but its biomarker value remains unconfirmed.
Extracellular matrix metalloproteinase induce (EMMPRIN)	(Rossi et al. 2017)	15 horses in control group; 17 with mEA; 12 with sEA	sEA	↑ (expression)	No respiratory function testing was performed, limiting objective assessment of respiratory distress and preventing clear differentiation between mEA and sEA. In addition, the study included client-owned horses kept under various stable conditions and 10 ponies, although the similar proportion of ponies across groups made a major effect on the results unlikely.	EMMPRIN protein was detected in BALF cells of all horses, but its expression was highest in horses with sEA and correlated positively with airway neutrophilia, MMP-2 and MMP-9 protein expression, and MMP-9 activity. These findings suggest that EMMPRIN is associated with neutrophilic airway inflammation and matrix remodelling in EA, although its clinical diagnostic value remains to be established.
Endothelin 1 (ET-1)	(Costa et al. 2009)	6 horses in control group; 6 with sEA	sEA	↑	High inter-individual variability in ET-1 concentrations and possible carry-over effects of prior bronchodilator or corticosteroid treatment.	ET-1 should be regarded primarily as a marker of local pathophysiological activity in EA, particularly airway inflammation and bronchoconstriction, rather than as a validated diagnostic biomarker. Although its concentration may increase during sEA exacerbation, its clinical diagnostic value remains unconfirmed, and it currently has no established role in the diagnosis or phenotypic classification of EA.
Hyaluronic acid (HA)	(Höglund et al. 2024)	15 horses in control group; 10 horses with neutrophilic mEA; 5 with sEA	sEA	↑	The study was limited by grouping based on airway cytology rather than the full clinical picture, classification restricted to neutrophilic airway inflammation despite mixed inflammatory patterns in some horses, uncontrolled environmental aspects such as stabling and transport, possible misclassification of horses in remission as controls, a small number of endobronchial biopsy (EBB) samples, HA molecular size assessment performed only in pooled samples precluding statistical analysis, and the lack of BALF dilution correction.	HA was significantly higher only in sEA, suggesting limited value for detecting neutrophilic mEA and a stronger association with inflammation severity than with EA presence alone. Its clinical diagnostic value remains unconfirmed, and it currently has no established role in the diagnosis or phenotypic classification of EA.
			neutrophilic mEA	↔		
Neutrophil extracellular traps (NETs)	(Janssen et al. 2022)	12 horses in control group; 14 with sEA; 7 with mEA	sEA	↑	A limitation of the study was the relatively small sample size, particularly for horses with mEA, which also restricted subgroup analyses within mEA. Three NETs detection methods differed in specificity, with cfDNA being the least specific because it may also reflect cell damage or death rather than NETs alone.	BALF NETs were increased specifically in horses with sEA, whereas they were minimal or undetectable in mEA and in healthy controls. NETs correlated positively with BALF neutrophil percentage and clinical severity, and showed diagnostic potential for identifying sEA. Among the evaluated methods, MPO-DNA complexes had the best predictive performance for sEA (AUC 0.906), followed by cfDNA (AUC 0.758) and confocal NETs area quantification (AUC 0.712).
	(Meiseberg et al. 2025)	8 horses in control group; 7 with sEA; 11 with mEA (7 with moderate EA; 4 with mild EA)	sEA	↑ (NET-activated cells)	A limitation was that the study did not distinguish sEA in exacerbation from sEA in remission, which may have obscured stage-dependent differences in NET-related mechanisms.	NET dysregulation increased with EA severity. BALF contained more NET-activated cells in sEA, and blood neutrophils from sEA horses showed enhanced ex vivo NET formation, supporting a link with disease severity rather than a robust stand-alone diagnostic marker.
cfDNA (component of NETs)	(Cooper et al. 2025)	19 horses in control group; 18 with sEA; 14 with mastocytic mEA; 12 with neutrophilic mEA	sEA	↑	A limitation of the study was that cfDNA is a non-specific marker that may derive not only from NETosis but also from apoptosis or necrosis.	BALF cfDNA was significantly increased in sEA compared with healthy horses and mastocytic mEA, supporting its potential as a biomarker of sEA. However, it did not clearly distinguish neutrophilic mEA from the other groups. Its clinical diagnostic value remains unconfirmed, and it currently has no established role in the diagnosis or phenotypic classification of EA.
Respiratory metabolites	(Bazzano et al. 2020)	6 horses in control group, 6 horses with sEA	sEA	Formate ↑	A limitation of the study was the small sample size.	Among the identified BALF metabolites, myo-inositol and glycerol were significantly decreased, whereas formate and isopropanol were increased in sEA. These findings suggest that BALF metabolite profiles reflect disease-associated airway biochemical changes and may have biomarker potential, particularly for myo-inositol. Its clinical diagnostic value remains unconfirmed, and it currently has no established role in the diagnosis or phenotypic classification of EA.
				Myo-inositol ↓		
				Glycerol ↓		
				Isopropanol↑		
Allergen-specific antibodies	(Schmallenbach et al. 1998)	3 horses in control group, 16 with sEA	sEA	IgE and IgG specific for *Aspergillus fumigatus* ↑	A limitation of the study was the small sample size of the control group relative to the affected group.	BALF *Aspergillus fumigatus*-specific IgE and IgG were significantly increased in sEA-affected horses. These findings suggest potential usefulness as local airway biomarkers of *Aspergillus*-related sEA. Its clinical diagnostic value remains unconfirmed, and it currently has no established role in the diagnosis or phenotypic classification of EA.
	(Jentsch et al. 2024)	18 horses in control group; 20 horses with mEA; 24 horses with sEA	mEA/sEA	IgA and IgG1 specific for *Aspergillus fumigatus* ↑ (binding)	The study was limited by substantial heterogeneity within the mEA group and by single-time-point sampling, which precluded longitudinal assessment of immunoglobulin dynamics.	*Aspergillus fumigatus*-binding IgA and IgG1 were increased in EA. This suggests potential usefulness as local airway biomarkers. Its clinical diagnostic value remains unconfirmed, and it currently has no established role in the diagnosis or phenotypic classification of EA.
				IgE specific for *Aspergillus fumigatus* ↔ (binding)		*Aspergillus fumigatu*s-binding IgE did not differentiate asthmatic and healthy horses. Current support for their role as candidate EA biomarkers remains limited.
	(Wyler et al. 2023)	39 healthy horses; 44 horses with EA	mEA/sEA	Allergen-specific IgE (panel; 17/18 analytes) ↑	A limitation of the BALF analysis was that sample filtration, although necessary for processing, may have led to loss of allergen-specific IgE trapped in mucus. The asthmatic horses in this study were not divided by asthma phenotype, so potential differences among subtypes could not be assessed.	BALF allergen-specific IgE was increased for most significant analytes (17/18) and showed moderate diagnostic performance (AUC 0.751), performing better than serum for diagnostic accuracy.
Cyclooxygenase products	(Gray et al. 1989)	5 horses in control group; 5 horses with sEA	sEA	Thromboxane B₂ (TXB₂); 6-keto-PGF_1α_; 9α,11β-PGF_2_ ↔	Only stable metabolites of cyclooxygenase products were measured, which may not have fully reflected rapid local changes in the active parent mediators within the airways	BALF concentrations of TXB₂, 6-keto-PGF1α and 9α,11β-PGF₂ did not differ significantly between heaves-affected and control ponies. Current support for their role as candidate EA biomarkers remains limited.
Fatty acid (FA)	(Höglund et al. 2023)	15 horses in control group; 10 with mEA; 5 with sEA	sEA	20:3n-6 (dihomo-γ-linolenic acid; DGLA) ↑	Individual differences in diet and the relatively small number of samples may have increased variability in FA measurements and lowered the predictive accuracy of the models.	In BALF, horses with EA showed altered FA composition. Increased 20:3n-6 was the most consistent change linked to airway inflammation. Overall, BALF FA profiles reflected inflammatory status. However, their accuracy was insufficient for reliable classification of uncategorized samples. In BALF-derived EV, horses with sEA showed decreased 16:0. They also increased 20:3n-6 and 20:5n-3. These findings indicate that severity is associated with alterations in lipid mediator cargo. Their clinical diagnostic value remains unconfirmed, and they currently have no established role in the diagnosis or phenotypic classification of EA.
				16:0 (palmitic acid) ↓		
				20:5n-3 (EPA) ↑		
Phospholipids	(Christmann et al. 2022)	30 horses in control groups; 12 with sEA; 8 with neutrophilic mEA; 10 with eosinophilic mEA	sEA	total phospholipid content ↓ and composition changes (phosphatidylglycerol (PG) ↓; cyclic phosphatidic acid (cPA) ↑)	Incomplete group matching beyond age. Small sample sizes within each group due to stringent selection criteria and limited sample availability across institutions. No correction for multiple comparisons (multiple testing). Low BALF neutrophilia, particularly in mildly asthmatic horses, because a 5% cutoff was used to distinguish healthy from asthmatic horses.	Surfactant phospholipid content and composition were significantly altered mainly in sEA, whereas only minor compositional changes were detected in neutrophilic mEA and no major phospholipid abnormalities were reported in eosinophilic mEA. This indicates biological relevance at the subtype level, but its biomarker value remains unconfirmed.
			neutrophilic mEA	total phospholipid content ↔ composition changes (phosphatidylglycerol (PG) ↓; cPA ↑)		
			eosinophilic mEA	total phospholipid content ↔ composition changes (phosphatidylglycerol (PG) ↔ cPA ↔)		
Procalcitonin (PCT)	(Barton et al. 2016a)	15 horses in control group;; 21 with sEA; 16 with mEA; 12 with CIP	mEA/sEA	↑	A key limitation was that most BALF PCT concentrations were below the ELISA’s validated working range for this matrix.	PCT concentrations were increased in horses with chronic respiratory disease, particularly in sEA and mEA, but did not differentiate reliably between the different chronic pneumopathy groups. Its diagnostic performance remains to be established.
FKBP5	(Riihimäki et al. 2023)	8 horses in control group; 11 horses with mEA	mEA	↑ (expression)	A limitation of the study was that FKBP5 was identified only at the transcriptomic level in BALF cells by scRNA-seq, without protein-level validation or assessment of diagnostic performance.	FKBP5 was markedly upregulated in BALF cells from horses with mEA, particularly in mast cells, strongly supporting its potential as a robust transcriptomic biomarker candidate and a reliable indicator of altered glucocorticoid responsiveness. Its clinical diagnostic value remains unconfirmed, and it currently has no established role in the diagnosis or phenotypic classification of EA.

**Fig. 2:**
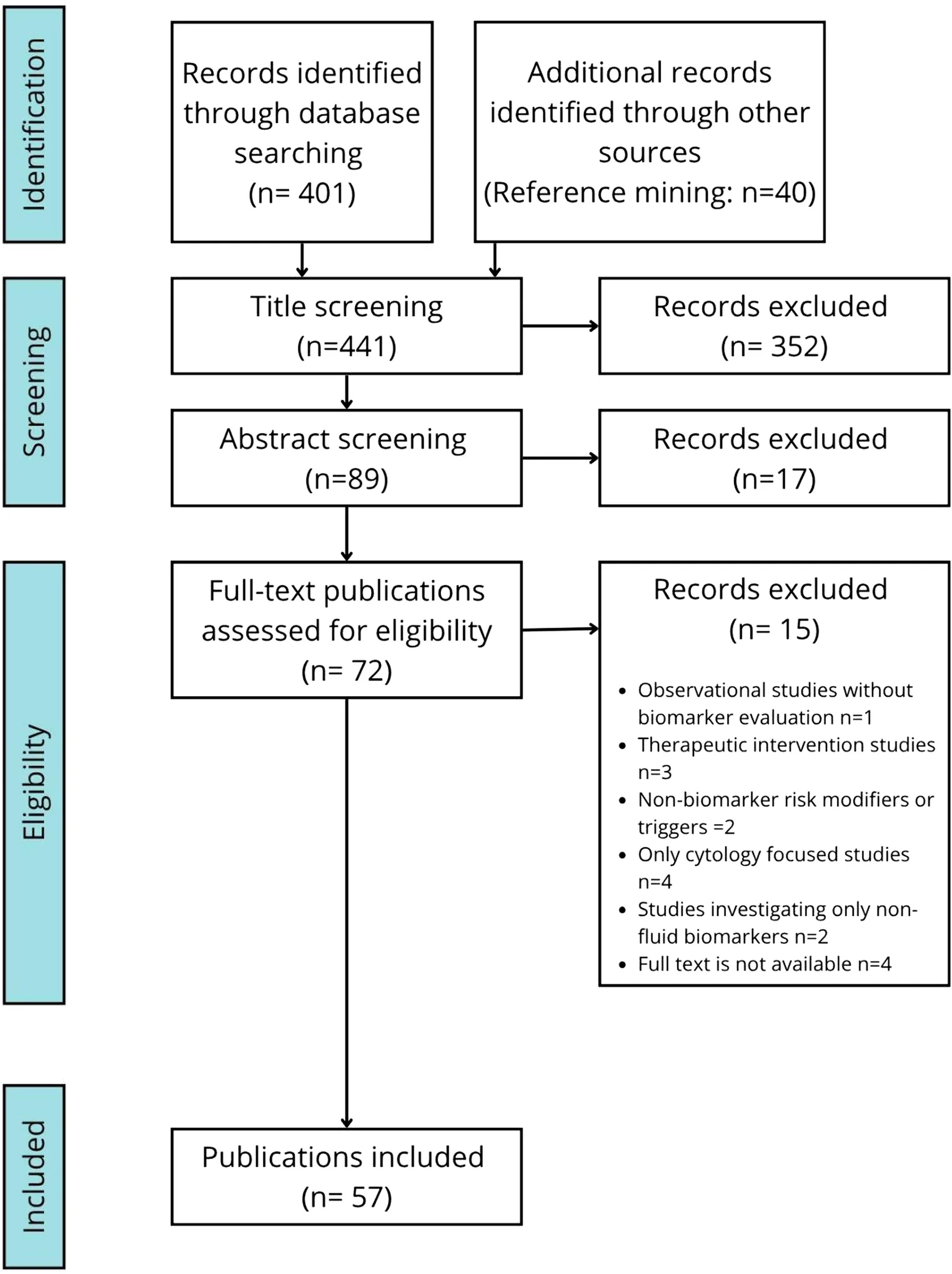
PRISMA flow diagram detailing the database searches, the number of titles and abstracts screened, and the full texts retrieved

Studies were eligible if they included horses diagnosed with mEA or sEA and reported quantifiable measurements of biomarkers in blood or BALF samples. Comparisons had to include either healthy controls or comparisons between mEA and sEA phenotypes. Reviews, opinion pieces, conference abstracts, experimental studies without biomarker measurements, and non-equine studies were excluded. Searches were performed in PubMed and Scopus using combinations of keywords related to the species (“equine”, “horse”, “pony”), the disease (“asthma”, “heaves”, “RAO or recurrent airway obstruction”, “IAD or inflammatory airway disease”, “COPD or chronic obstructive pulmonary disease”, “respiratory disease”, “airway disease”, “pneumopathy”, “respiratory inflammation”), and the biological matrix (“serum”, “plasma”, “blood”, “bronchoalveolar lavage fluid” or “BALF”,) and “biomarker”. This combined search string ensured coverage of studies regardless of whether the specific matrix or disease term was explicitly stated in the title. Google Scholar was primarily used to identify additional records through citation tracking. Additional references were retrieved through manual screening of the reference lists of relevant articles. The last search was conducted on 30.01.2026. To ensure a comprehensive literature coverage, reference mining was conducted. Only studies published in English were included. After removing duplicates, titles and abstracts were screened to identify potentially eligible studies. Full-text articles were then reviewed based on the predefined inclusion criteria. The search strategy and study selection results are presented in Fig. 2. For each eligible study, data were systematically extracted, including: citation, type of biological sample (blood or BALF), asthma phenotype (mEA or sEA), biomarker(s) investigated, direction of change (↑ increase, ↓ decrease, ↔ no change), group sizes, and key methodological notes or limitations. The extracted data were summarised in two separate tables: Table 2 (blood-derived biomarkers) and Table 3 (BALF biomarkers).

No meta-analysis was performed. No review protocol was registered for this study. No formal risk-of-bias or quality tool was used. Potential bias sources were considered qualitatively, regarding study design, sample size, and result consistency. Results were summarised narratively. They were grouped by matrix and biomarker type, with a focus on repeatability and diagnostic potential.

## Results

In total, 57 unique studies were identified, corresponding to 68 table entries, as 11 studies contributed data to both blood- and BALF-based analyses. 25 entries described blood-based biomarkers (14 unique studies and 11 studies that also evaluated BALF), whereas 43 entries described biomarkers measured in BALF or other matrices (32 unique studies and the same 11 shared with Table 2). Because several biomarkers were evaluated in more than one study, Tables 2 and 3 were structured primarily by biomarker rather than by study.

Most investigated biomarkers have been evaluated solely for statistical significance. In contrast, only a minority have been assessed using formal diagnostic performance measures. The most well-established potential biomarkers in blood are haptoglobin, surfactant protein D (SP-D), and secretoglobin (SCGB). Particularly when assessed in combination rather than individually. Allergen-specific IgE profiles have also been evaluated for diagnostic performance, although available data suggest they are more informative when assessed in BALF than in serum. Some biomarkers showed statistically significant differences between horses with EA and healthy controls, as well as between individual phenotypes. However, their diagnostic value has not yet been confirmed. This group contains serum amyloid A (SAA), thromboxane B₂ (TXB₂), endothelin-1 (ET-1), 8-hydroxy-2-deoxyguanosine (8-OHdG), leucine‑rich repeats and calponin‑homology domain‑containing 4 (Lrch4), microtubule‑associated serine/threonine‑protein kinase 4 (MAST4), microRNA (miRNA), redox biomarker trolox equivalent antioxidant capacity (TEAC) and ferric reducing antioxidant power (FRAP), neutrophil cellular cholesterol and procalcitonin (PCT). Further studies are therefore needed to validate these candidates and to establish standardised methods and clinically meaningful thresholds before their diagnostic value as EA biomarkers can be confirmed. A further group consists of biomarkers whose values did not change significantly between groups, nor do their blood concentrations reflect lower airway inflammation usefully. This category includes several serum cytokines (IL-10, CCL2, IFN-γ, IFN-α, IL-2) as well as C-reactive protein (CRP), neutrophil gelatinase-associated lipocalin (NGAL), 25-hydroxyvitamin D (25(OH)D), myostatin (MSTN), hyaluronic acid (HA), soluble triggering receptors expressed on myeloid cells-1 (sTREM-1), circulating immune complexes (CICs), dexamethasone-induced protein (DEXI), and the redox biomarkers glutathione reductase (GSHred), advanced oxidation protein products (AOPP), and superoxide dismutase (SOD).

BALF biomarkers have more pathophysiological relevance than blood-based markers because they reflect local airway inflammation. Biomarkers for which diagnostic performance values have been reported in BALF are neutrophil extracellular traps (NETs) related markers, especially myeloperoxidase (MPO) DNA complexes, NGAL and allergen-specific IgE panel. The group of biomarkers showing statistically significant changes in EA, while still requiring further investigation to determine their diagnostic usefulness, includes: C-X-C motif chemokine ligand (CXCL-8 or IL-8), arachidonate metabolites such as prostaglandin F (PGF), prostaglandin E2 (PGE2); histamine, SCGB, transferrin (TF), redox biomarker (TEAC, AOPP, SOD), myristoylated alanine-rich C kinase substrate (MARCKS), MPO, cytokines (IFN-γ, IL-1β, IL-4, IL-10), neurokinin A (NKA), CXCL13, KIT proto‑oncogene receptor tyrosine kinase (c‑KIT/CD117), matrix metalloproteinases/tissue inhibitors of metalloproteinases (MMPs/TIMPs: MMP-2, TIMP-1, TIMP-2, MMP-9, MMP-8), extracellular matrix metalloproteinase inducer (EMMPRIN), ET-1, HA, respiratory metabolites, fatty acid (FA), phospholipids, PCT, and FKBP5. BALF biomarkers with unchanged or non-significant levels include: TNF-α, IL-17 A, arachidonate metabolites such as 6-keto-prostaglandin F1 alpha (6-keto-PGF1α), TxB2, leukotriene B4 (LTB4), prostaglandin D2 (PGD2), leukotriene C4 (LTC4); redox biomarkers such as GSHred, FRAP; and cyclooxygenase products: TXB₂, 6-keto-PGF1α, 9α,11β-PGF_2_.

Three included studies also assessed biomarkers in matrices other than blood or BALF. One studied redox markers in tracheal washes (TW), saliva, serum, and BALF. Another combined BALF with exhaled breath condensate metabolomics. A third analysed tissue biopsies from endobronchoscopy. These sample types were presented narratively rather than in separate tables because of their small numbers and different methods.

The reviewed studies confirm that research on biomarkers of EA is affected by several recurring methodological limitations. The most frequently reported issue is the small sample size. Low numbers of horses in study groups reduce statistical power, increase the risk of error, and limit the generalizability of findings to the wider equine population. Another important limitation is the lack of longitudinal studies following horses over time, which also makes it difficult to assess biomarker dynamics in relation to disease course, seasonality, or treatment response. Defining and selecting appropriate control groups remains another major challenge. Horses in clinical remission or with subclinical disease may be misclassified. This can confound between-group comparisons. Asthma classification was often inconsistent or incomplete. The lack of stratification by phenotype and endotype may further affect results. Findings are also strongly influenced by environmental factors and concurrent diseases. Both may substantially affect biomarker concentrations and complicate interpretation. This is particularly relevant in field studies. Differences in housing, feeding, allergen exposure, exercise, or comorbid conditions may act as important confounders. In addition, many studies lack proper validation and methodological standardisation. Most studies report only statistical differences between groups. Only a minority provide formal diagnostic performance measures, such as sensitivity, specificity, AUC, or cut-off values. This substantially limits clinical interpretability. Statistical significance alone does not indicate whether a biomarker is sufficiently accurate for practical diagnostic use. Additional variability arises from preanalytical and matrix-related factors, especially in BALF-based studies. Differences in recovery volume, uncontrolled dilution, filtration, and the absence of internal normalisation may substantially affect analyte concentrations.

## Discussion

BALF and blood are currently the two main biological matrices investigated for EA biomarkers. Other sample types, including saliva, EBC, and TW, have also been evaluated, but in a limited number of studies (Bazzano et al. 2020; Hansen et al. 2025; Cooper et al. 2025). BALF cytology is considered the gold standard for diagnosing and studying EA, as it reflects local inflammatory processes within the airways. BALF analysis enables the identification of both cellular and molecular markers of the disease. In recent years, there has been increasing interest in biomarkers in this fluid, which may complement classical cytology by improving diagnostic sensitivity and specificity and enabling monitoring of disease activity and phenotypes. Research has focused on various potential biomarkers, such as cytokines, acute-phase proteins, oxidative stress mediators, and proteomic components, which may serve as diagnostic and therapeutic targets. The investigated biomarkers of EA in BALF are summarised in Table 3. Although blood biomarkers are less specific for airway disease than BALF, they are minimally invasive and suitable for repeated sampling. Peripheral blood biomarkers reflect systemic indicators of inflammation and immune activation. They may provide supportive information for diagnosis, prognosis, or treatment monitoring. Table 2 presents potential blood biomarkers investigated in EA.

### Acute phase protein (APPs)

APPs, including haptoglobin, SAA, and CRP, have been evaluated as markers of systemic inflammation in horses with lower airway disease. Some studies have explored combinations of biomarkers to improve diagnostic sensitivity and specificity. Multimarker approaches may better represent the complex pathophysiology of EA than single analytes.

In Lavoie-Lamoureux et al. (2012) study, experimental exposure to antigens led to elevated serum haptoglobin and SAA levels in horses with sEA. Asymptomatic sEA-affected horses exhibited haptoglobin concentrations approximately 2.5 times higher than healthy controls. Although control horses also showed increased serum haptoglobin following hay exposure, their levels returned to baseline within 30 days, in contrast to the persistent elevation observed in sEA-affected horses. The study further reported up to a 50-fold increase in SAA in sEA-affected horses after 7 days of antigen exposure, a response seen in only one control horse. No statistically significant differences were found in CRP levels. Bullone et al. (2015) later confirmed the diagnostic value of measuring both serum haptoglobin and SP-D in horses with mEA. This combination of biomarkers had the highest sensitivity and specificity among the four analytes tested. The study also measured SAA, which was 3.5 times higher in the control group. sTREM-1 showed no significant difference between groups. Further evidence comes from a study evaluating serum haptoglobin, SP-D, and SCGB in horses with neutrophilic mEA. The study showed these markers can serve as useful diagnostic tools in animals with clinical signs of lower airway disease and neutrophilic inflammation. Diagnostic performance varied by marker set. The combination of SP-D, haptoglobin, and SCGB achieved 100% specificity but only moderate sensitivity (57%). Incorporating the scoring system of a Horse Owner Assessed Respiratory Signs Index (HOARSI) = 2 further improved diagnostic validity and maintained 100% specificity across several marker combinations (Gy et al. 2019). Recent data show that high serum SP-D levels mainly occur in sEA. This supports the idea that SP-D and related acute-phase responses are most helpful in diagnosing severe, neutrophil-predominant disease rather than across all EA phenotypes. Christmann et al. (2022) found that SP-D levels were higher only in horses with sEA compared to controls, with no change in milder types (neutrophilic and eosinophilic mEA). Serum haptoglobin was also higher in sEA than in controls and did not change in mEA. The sum of SP-D and haptoglobin was higher in sEA, suggesting that this may aid in diagnosing severe disease. Richard et al. (2012) demonstrated that serum SP-D concentrations were significantly higher in horses with mEA compared to controls, both at rest and 60 min post-exercise. Physical exertion did not significantly affect SP-D levels within either group, and pre- and post-exercise values were strongly correlated. No association was found between serum SP-D and BALF cytology, nor were there differences between inflammatory phenotypes (neutrophilic vs. mastocytic/eosinophilic). These findings suggest that mEA is associated with a moderate but measurable increase in circulating SP-D, most likely reflecting alveolar epithelial damage and increased alveolar-capillary barrier permeability during inflammation. However, the authors emphasised the need for cautious interpretation due to the use of a human-derived enzyme-linked immunosorbent assay (ELISA) kit and the lack of species-specific assay validation. However, acute-phase proteins may not serve as reliable markers of lower airway inflammation, as shown by Leclere et al. (2015). In that study, three proteins (SAA, haptoglobin, and CRP) were measured in racehorses with poor performance compared to those with mEA. There were no important differences in the levels of these proteins between the groups. The authors emphasised that elevated protein levels can be caused by other factors, such as training in difficult conditions. Also, protein levels did not differ between horses with different lung cell types in their BALF, except for lower CRP levels in horses with neutrophilic inflammation. In Lee et al. (2022), serum SAA increased after antigen challenge in sEA, indicating systemic inflammation, but this was not statistically significant in this small group.

APPs, especially haptoglobin, SAA, and to some extent SP-D, were associated with EA. Their diagnostic value varied by disease phenotype and severity. The most promising results came from multimarker approaches. Combining haptoglobin and SP-D, and in some studies SCGB, improved diagnostic specificity. However, individual acute-phase proteins remain of limited usefulness because nonspecific factors, such as exercise, environment, or systemic inflammation, can affect their concentrations. Data also suggest APPs and SP-D are more informative in severe, neutrophilic forms of EA, particularly sEA, than in mild forms.

### Neutrophil-associated markers

Given the central role of neutrophils in EA pathophysiology, substantial attention has also been directed to neutrophil-associated mediators in BALF. One example is NGAL. Studies in humans have demonstrated elevated blood NGAL levels in individuals with asthma, with concentrations linked to clinical parameters and suggesting involvement in airway remodelling (Kawagoe et al. 2021). For this reason, NGAL has been proposed as a potential biomarker for neutrophilic phenotypes of EA. Recent findings show that its concentration was low in healthy horses and increased with disease severity. Importantly, NGAL levels in BALF differed significantly between mEA and sEA, as well as between horses with a tracheal mucus score (TMS) ≤ 2 and those with TMS > 2 (scale 0–5). In contrast, no association was found between serum NGAL concentration and the diagnosis of EA, nor was there a correlation between serum NGAL and BALF NGAL concentrations (Hansen et al. 2024).

NETs may also play a role in EA pathophysiology. NET levels showed a positive correlation with both EA severity and the proportion of neutrophils in BALF. Based on these associations, NETs were proposed as a potential predictive biomarker for sEA and for distinguishing it from mEA (Janssen et al. 2022). Meiseberg et al. (2025) further demonstrated that NET biology is dysregulated in a severity-dependent manner in EA. Specifically, the proportion of NET-activated cells in BALF increased with disease severity, whereas cell-free NET markers showed limited discriminatory value. At the systemic level, severely affected horses exhibited an increased NET-forming capacity of circulating neutrophils and elevated circulating anti-neutrophil cytoplasmic antibodies (ANCA). These changes were accompanied by a severity-dependent decrease in neutrophil cellular cholesterol, which correlated with enhanced NET formation and hypoxia, supporting the presence of a cholesterol-associated inflammatory component in disease progression. Consistent with the concept of NET-related dysregulation, Cooper et al. (2025) reported significantly increased cfDNA concentrations in both BALF and TW supernatant in horses with sEA using a portable Qubit fluorometer, highlighting the translational potential of cfDNA-based assays for near-stall diagnostics. Nevertheless, it is important to note that cfDNA was less informative for distinguishing milder phenotypes.

### Oxidative stress indicators

Consistent with the prominence of neutrophils, oxidative stress has been directly demonstrated in EA. Early work showed rapid neutrophil recruitment to the lungs of allergic horses after antigen exposure (Fairbairn et al. 1993). More recently, Hansen et al. (2025) validated and quantified five redox biomarkers serum, saliva, TW fluid, BALF in healthy horses and in animals with neutrophilic mEA, mastocytic mEA, and sEA. These included two indicators of total antioxidant capacity (TEAC, FRAP), two antioxidant enzymes (SOD, GSHred), and one marker of oxidative protein damage (AOPP). Serum GSHred, AOPP and SOD showed no significant differences between healthy horses and animals with the EA. This indicates a limited degree of systemic redox imbalance across all studied groups. In contrast, the mastocytic mEA group had significantly higher serum FRAP and TEAC concentrations compared to neutrophilic mEA, sEA, and healthy control groups. Additionally, stallions had higher serum FRAP values compared to geldings within the study population. In BALF samples, concentrations of TEAC, SOD, and AOPP were significantly reduced with increasing asthma severity, and these markers correlated negatively with BALF neutrophil counts, especially in the neutrophilic groups. GSHred did not show differences among groups in BALF, and FRAP variation among groups was minimal. In TW, biomarker results were variable; for example, AOPP was unexpectedly higher in sEA horses compared to controls. The analysis confirmed that oxidative stress is most pronounced in neutrophilic EA, whereas mastocytic mEA showed only limited changes in redox status, suggesting that different mechanisms predominate in these groups. Significant negative correlations were also observed between neutrophil counts and the saliva markers SOD, GSHred, and AOPP, but changes in saliva and serum were generally less marked than those seen in BALF. This evidence shows that BALF provides the clearest group differences for assessing redox imbalance in EA. These findings suggest that antioxidant depletion may be associated with disease severity and that TEAC, SOD, and AOPP could be further explored as markers of neutrophilic asthma in horses.

Another systemic oxidative stress indicator that may serve as a potential blood biomarker in EA is 8-OHdG. Niedźwiedź et al. (2015) evaluated 8-OHdG serum concentrations in horses with sEA. Their study assessed biomarker levels following 48 h of exposure to mouldy hay. Serum 8-OHdG was detected in all animals. However, levels were substantially higher in sEA cases than in controls, reflecting an almost 10-fold increase. This elevation is consistent with oxidative stress. Disruption of the oxidant–antioxidant balance favours the modification of deoxyguanosine to 8-OHdG within DNA. Importantly, current evidence suggests that such DNA damage occurs alongside sEA as a downstream consequence of chronic airway inflammation, rather than as its initiating cause. The observed redox imbalance also supports the idea that strengthening antioxidant defences could provide therapeutic benefit. Further studies have proposed developing novel pharmacological interventions targeting inflammatory mediators as a potential treatment avenue.

Among other oxidative-stress mediators, particular attention has been given to MPO. Art et al. (2006) showed that in horses with sEA, exposure to mouldy hay significantly increased BALF MPO concentrations, which remained elevated after clinical remission compared with healthy controls, indicating persistent subclinical neutrophil activation and ongoing oxidative stress. BALF MPO concentrations correlated positively with neutrophil counts during both exacerbation and remission, supporting MPO as a biomarker of neutrophilic inflammation in EA.

Cell-associated markers may complement soluble BALF biomarkers. They capture intracellular signalling pathways that drive neutrophilic inflammation and oxidative injury. Conley et al. (2025) showed that (MARCKS) protein is elevated in BALF cell lysates from horses with asthma compared with healthy animals. This identifies MARCKS as a novel cell‑associated marker of the neutrophilic phenotype of EA. The Myristoylated N-terminal Sequence (MANS) peptide blocks MARCKS. It reduced zymosan‑induced reactive oxygen species (ROS) production in alveolar macrophages and peripheral‑blood neutrophils. However, it did not significantly affect the release of the cytokines TNFα or IL‑8. Thus, MARCKS appears more valuable as a mechanistic or therapeutic target linked to oxidative stress than as a soluble cytokine biomarker. Combining cell‑associated markers like MARCKS with traditional soluble BALF biomarkers may more effectively capture the intracellular signalling pathways that drive neutrophilic inflammation and oxidative injury.

### Cytokines, chemokines and inflammatory mediators

Numerous studies have evaluated soluble mediators, such as cytokines and chemokines, which better reflect the inflammatory phenotype of EA. Horses with sEA show higher BALF concentrations of CXCL-8, TNF-α, and IFN-γ than healthy individuals (Woodrow et al. 2023). However, findings on TNF-α are inconsistent. Some studies reported elevated TNF-α levels (Richard et al. 2014; Woodrow et al. 2023), whereas others found decreased levels during natural exacerbations (Riihimäki et al. 2008c; Montgomery et al. 2018). Reports on TNF-α mRNA expression are also conflicting, with some studies showing upregulation (Hughes et al. 2011) and others no significant change (Riihimäki et al. 2008c). These variations likely stem from differences in methods, such as mRNA versus protein analysis, disease stage (acute or chronic), and study populations (experimentally induced versus natural disease) (Montgomery et al. 2018). Studies also note clear distinctions in cytokine/chemokine profiles and BALF cell composition between asthmatic phenotypes and healthy horses (Richard et al. 2014; Woodrow et al. 2023). Larger studies with standardised methods could help identify reliable biomarkers, supporting the subclassification of EA by BALF cytology: eosinophilic mEA, neutrophilic mEA, mastocytic mEA, or sEA. These subtypes may require tailored management and treatment, underscoring the need for phenotype-specific research (Woodrow et al. 2023).

IL-8 is recognised as a key biomarker of neutrophilic inflammation within the equine airway cytokine network. Numerous studies have shown that in horses with severe respiratory conditions, there is an elevated expression of the IL-8 gene in bronchoalveolar cells, accompanied by a marked increase in IL-8 protein concentration in BALF (Franchini et al. 2000; Giguère et al. 2002; Ainsworth et al. 2003, 2006, 2009; Pietra et al. 2007; Riihimäki et al. 2008a; Padoan et al. 2013; Hansen et al. 2014). Franchini M. (2000) reported significantly higher BALF IL-8 concentrations and neutrophil chemotactic activity in horses with sEA compared with healthy controls, and IL-8 levels further increased in asymptomatic affected horses after dust exposure induced by a dietary change from silage to hay. IL-8 was also evaluated by immunohistochemistry, along with NKA, in the bronchial epithelium of horses with sEA at different disease stages. While no differences in IL-8 expression were observed between the various clinical phases of asthma, a significant difference in epithelial immunoreactivity was confirmed between healthy and asthmatic horses. In horses with sEA, IL-8 expression appeared intense, often focal, and confined to individual epithelial cells, whereas in healthy horses, it was more diffuse and markedly weaker (Morini et al. 2021). While most studies report elevated IL-8 in sEA, findings are not entirely consistent across different EA phenotypes. Some studies report no significant increase in IL-8 mRNA in horses with mild neutrophilic inflammation (Lavoie et al. 2011), and the response may be age-dependent, with older healthy horses showing naturally decreased IL-8 expression (Hansen et al. 2014). Additionally, differences between mRNA expression and protein levels have been noted (Padoan et al. 2013), suggesting complex post-transcriptional regulation of IL-8 in equine airways.

In addition to pro-inflammatory cytokines, regulatory cytokines such as IL-10 have also been investigated as prospective biomarkers in EA. In a study evaluating serum IL-10 in horses with sEA before and after hay exposure, no significant changes were observed. Likewise, no statistically significant differences in serum CCL2 or IFN-γ concentrations were observed between conditions. IFN-α and IL-2 remained below detection limits in all samples (Lavoie-Lamoureux et al. 2012). Consistent with these findings, Hamouzová et al. (2023) found no statistically significant differences in plasma IL-10, IFN-γ, IL-4, and IL-17 between horses with mEA and sEA. Beekman et al. (2012) reported significantly higher IL-10 mRNA expression in BALF from horses with mEA compared with controls. However, after stratifying by inflammatory phenotype (neutrophilic mEA and mastocytic mEA), this increase was not statistically significant. In the same study, IL-10 expression correlated positively with the percentage of neutrophils in BALF. In summary, IL-10 levels differ by sample type and inflammatory phenotype, making IL-10 an unreliable standalone biomarker for EA. The key takeaway is that IL-10 should only be interpreted within the context of phenotype-stratified, paired protein–mRNA assessments for accurate evaluation of EA.

In another study, IL-1β and KIT (CD117) were identified as promising biomarkers for differentiating mEA subtypes (Davis and Sheats 2021). Building on these outcomes, IL-1β was subsequently evaluated and correlated with BALF neutrophilia; however, as with IL-17 A, its level did not differ between subtypes. In contrast, IL-4 was higher in the mixed subtype and linked to mast cells. Additionally, Th1 cytokines (IL-2, IFN-γ) showed no meaningful associations (Lo Feudo et al. 2024).

Karagianni et al. (2024) provide a framework for interpreting discrepancies across cytokine-focused studies in mEA. They demonstrated that BALF cytology reflects distinct immunological endotypes. Neutrophil-predominant samples were enriched for pathways related to neutrophil chemotaxis and phagocytosis. In contrast, mastocytic samples were associated with hypersensitivity responses, fibrogenesis, and oxidative stress. This confirms that immune signalling is strongly endotype-dependent. The authors emphasise that cytokine “single-analyte” approaches often yield inconsistent results. BALF cytology is highly variable in practice (pure, mixed, or overlapping infiltrates), leading to convergence of inflammatory pathways and limiting the interpretability of any single marker set.

It is also helpful to consider data from Riihimäki et al. (2008b). Although the study did not include horses with a confirmed diagnosis of EA, it provides important information on environmentally induced airway inflammation. Notably, it describes changes in cytokines, especially IL-6 and IL-10, in BALF. A seasonal study in racing Standardbreds found that winter stabling, with slightly higher dust and β‑glucan levels, led to more BAL neutrophils and much higher IL-6 mRNA. IL-10 responses were more variable, underscoring the context-dependent nature of regulatory cytokines.

Costa et al. (2009) evaluated ET-1, a potent bronchoconstrictive and pro-inflammatory peptide, in plasma and airway fluids. ET-1 concentrations increased during the exacerbation of seasonal sEA. This change was observed in both plasma and BALF. It also appeared in urea-corrected epithelial lining fluid. These results support ET-1 as an exacerbation-associated candidate biomarker rather than a stable marker in remission.

### Markers of extracellular matrix remodelling

In the study by Barton et al. (2015), the activities of MMP-2, MMP-8, MMP-9, TIMP-1 and TIMP-2 were evaluated in BALF from horses with chronic lower airway diseases, including sEA, mEA, and chronic interstitial pneumopathy (CIP). The results showed that MMP-2, TIMP-1, and TIMP-2 concentrations were significantly elevated in horses with sEA and mEA compared to healthy controls, whereas MMP-8 and MMP-9 were increased across all disease groups (Barton et al. 2015). Consistently, Hamouzová et al. (2023) reported that BALF MMP-9 was significantly higher in sEA than in mEA and was higher in neutrophilic/mixed cytology than in mastocytic profiles; BALF MMP-9 correlated positively with BALF neutrophil percentage and clinical score, additionally supporting MMP-9 as a severity- and neutrophilia-associated airway biomarker. Marked correlations were observed among MMP/TIMP levels, clinical findings, and BALF neutrophil percentages. Notably, the MMP-8/TIMP-1 ratio proved notably valuable, as it reflects the balance between tissue destruction (high ratio) and fibrosis formation (low ratio), allowing differentiation of disease severity and phases and may provide prognostic value in equine pneumopathies (Barton et al. 2015). In this study, MMP-2 protein levels were significantly higher in horses with neutrophilic mEA than in those with the mastocytic endotype (Davis and Sheats 2021). Beyond MMPs themselves, Rossi et al. (2017) identified EMMPRIN in BALF cells as a severity-associated candidate biomarker, with higher expression in horses with respiratory distress and positive correlations with BALF neutrophilia, MMP-2/−9 expression, and MMP-9 activity, supporting an upstream role in neutrophil-associated remodelling pathways. Significantly elevated levels of MMP-8 and MMP-13 in tracheal epithelial lining fluid (TELF) have also been reported in horses with sEA compared to healthy controls (Raulo et al. 2001). Barton et al. (2016b) showed that after 10 days of inhaled budesonide combined with environmental dust reduction, clinical improvement in sEA was accompanied by marked decreases in BALF MMP-2, MMP-9, TIMP-1, and TIMP-2 concentrations, and by improved MMP-8/TIMP ratios, supporting their usefulness as treatment-response biomarkers. Notably, MMP-9 and TIMP-2 were highlighted as particularly informative markers of clinical improvement.

Another extracellular matrix–related biomarker candidate is HA. In a prospective case–control study of naturally occurring neutrophilic EA, Höglund et al. (2024) found that HA was increased in BALF supernatant in asthmatic horses, associated with airway neutrophilia, mucus burden, and inversely correlated with arterial oxygenation (PaO₂). This links luminal HA to inflammation severity and impaired gas exchange. In contrast, plasma HA concentrations and bronchial tissue HA staining intensity did not differ between groups. Histological airway remodelling was seen mainly in severe neutrophilic airway inflammation, suggesting that BALF HA may reflect neutrophilic inflammation severity without paralleling structural airway changes in mild or moderate stages. Importantly, HA effects depend on molecular size: low-molecular-weight HA is generally pro-inflammatory, while high-molecular-weight HA is considered more protective. Thus, total HA concentration alone may not reflect its pathobiological role in EA.

### microRNAs

Further investigations focused on molecular biomarkers, including miRNAs. To detect possible serum miRNA biomarkers in EA, small RNAs were extracted from the serum of 34 healthy horses and 37 with EA. These samples went through next-generation sequencing (NGS), followed by both novel miRNA discovery and differential expression analysis. As a result, eleven miRNAs were found to be significantly differentially expressed between asthmatic and control groups. Furthermore, pathway analysis linked these miRNAs to processes critical in asthma, such as epithelial-to-mesenchymal transition (airway remodelling) and the phosphatidylinositol (3,4,5)-trisphosphate (PIP3) signalling pathway, which controls CD4 + T cell function. Notably, decreased levels of miR-128 and miR-744 indicated a change towards a Th2/Th17 immune response in sEA (Pacholewska et al. 2017). While individual miRNAs are well characterised, it is important to note that evidence suggests they act cooperatively within regulatory networks. For example, in human bronchial epithelial cells, inhibiting miR-18a, miR-27a, miR-128, and miR-155 increased IL-6 and IL-8 mRNA, and IL-6 secretion rose at the protein level, though IL-8 secretion was not statistically significant (Martinez-Nunez et al. 2014). Collectively, these findings provide valuable translational insights and may identify promising directions for future miRNA research in horses.

More recently, high-dimensional approaches have refined this immunological picture. Single-cell RNA sequencing (scRNA-seq) analysis of BALF cells has revealed the specific role of CXCL13 in sEA pathogenesis. The results suggest that sEA is mainly driven by a Th17 immune response, rather than a Th2 response. Monocytes play a key role in starting Th17 polarisation. This may happen through direct interactions with T cells. Monocytes in both lung and blood show higher CXCL13 expression, highlighting its potential as a biomarker for sEA. Asthmatic horses also showed more neutrophil activity and irregular T-cell gene expression. These findings support the use of the equine model for studying neutrophilic asthma in humans (Sage et al. 2024). Consistent with these scRNA-seq findings, BALF cell mRNA profiling further supports a heterogeneous but predominantly innate/Th17-driven response in EA (Lo Feudo et al. 2024). This concept is also supported by an earlier transcriptomic study of stimulated peripheral blood mononuclear cells, in which CXCL13 was among the most strongly upregulated genes in horses with sEA, particularly after hay dust extract stimulation. The authors suggested that CXCL13 may be produced by monocytes, macrophages and Th17 cells, reflecting the presence of a systemic immune component linked to this chemokine in sEA (Pacholewska et al. 2015).

In a recent scRNA-seq study of BALF cells, FKBP5 emerged as one of the most significantly upregulated genes in horses with mEA compared with healthy controls, with the strongest expression observed in mast cells. This finding identified FKBP5 as a likely transcriptomic biomarker candidate and suggested a possible link between mastocytic airway inflammation and altered glucocorticoid receptor regulation (Riihimäki et al. 2023).

### Allergen-specific and mast cell-related biomarkers

While neutrophilic and Th17-driven inflammation appears central in sEA, mast cell–related mechanisms and allergen-specific responses have also been explored. Histamine is a key mediator released by mast cells during allergic responses. It has been investigated as a potential biomarker of EA. In a study by Hare et al. (1999), pulmonary mast cells were isolated from the BALF of horses with sEA. These cells released significantly greater amounts of histamine in response to allergen challenge than cells from clinically healthy horses. This difference was evident both during clinical remission and during disease exacerbation. The greatest histamine release occurred after exposure to common hay-related fungi such as *Aspergillus fumigatus* and *Alternaria tenuis*. Although histamine reflects mast cell hyperreactivity in EA, its rapid degradation and low specificity limit its value as a standalone biomarker.

Antibody responses to environmental allergens, especially *Aspergillus fumigatus*, have also been investigated as possible biomarkers in EA. Early evidence indicated that horses with sEA had significantly increased IgE and IgG antibodies against both somatic and recombinant *A. fumigatus* antigens in BALF. No such differences were detected in serum. These outcomes pointed to the predominantly local character of the immune response. They also suggested that BALF antibody profiles may be useful for identifying *Aspergillus-*associated exacerbations of sEA. Moreover, immunoblot analysis of BALF identified four reproducible *A. fumigatus* antigens consistently recognised by both IgE and IgG. These were proposed as candidate biomarker targets (Schmallenbach et al. 1998). More recent data have only partly supported these observations. Specifically, Jentsch et al. (2024) reported significantly higher binding of *A. fumigatus*-specific IgA and IgG1 in BALF from horses with mEA and sEA compared with healthy controls. However, IgE levels and total immunoglobulin concentrations remained unchanged. Therefore, this suggests that allergen-specific antibody responses persist in the lower airways. As a result, the diagnostically relevant immunoglobulin classes may differ from those emphasised in earlier studies. A similar pattern manifested from protein microarray studies. In serum from horses with sEA, allergen-specific IgE profiling revealed a wider systemic sensitisation pattern dominated by latex, fungal, mite, and pollen proteins. Major contributors included Hev b 11, Hev b 6.02, Hev b 5.0101, and rAsp f 8. However, this signal was detectable only within a broader allergen panel and appeared to be influenced by environmental exposure. Therefore, serum allergen-specific IgE is more informative within a multiallergen profile than as a stand-alone biomarker (White et al. 2019). In contrast, BALF allergen-specific IgE patterns discriminated asthmatic horses from controls more effectively than serum profiles. The most informative responses involved fungal allergens, particularly *A. fumigatus*, as well as *Culicoides spp*. and latex-related proteins. Taken together, these data further support the view that allergen-specific antibody responses in EA are predominantly local rather than systemic (Wyler et al. 2023).

### Arachidonic acid metabolites

Among the earliest investigations exploring chemical compounds that could potentially serve as biomarkers of EA, Gray et al. (1989) evaluated arachidonic acid metabolites produced by the cyclooxygenase pathway. These metabolites are known to affect bronchial smooth muscle tone and inflammatory signalling. In this study, both plasma and BALF concentrations of TXB₂, 6-keto-PGF1α (marker for prostacyclin PGI₂), and 9α,11β-prostaglandin F₂α (marker for PGD₂) were measured in ponies with sEA and matched controls. Only plasma TXB₂ increased during acute airway obstruction. The prostaglandin metabolites remained unchanged in both compartments. Cyclooxygenase inhibition reduced TXB₂ levels but did not affect airway function. This indicates that these products are biomarkers of inflammation rather than causal mediators. The limited specificity of TXB₂ supports targeting alternative pathways, such as lipoxygenase metabolites, for diagnosis and therapy.

The study quantified prostaglandins (immunoreactive PGF, PGE2, PGD2, 6-keto-PGF1α as an index of PGI2, TxB2) and leukotrienes (LTB4, LTC4). Horses with sEA showed significantly higher concentrations of PGE2 and PGF2 than healthy animals. No group differences were observed for PGD2, 6-keto-PGF1α, TxB2, LTB4, or LTC4. PGD2, LTB4, and LTC4 were present at relatively high levels in clinically normal horses. Although the authors did not propose biomarker applications, their findings show that elevated PGE₂ and PGF levels may reflect airway inflammation and bronchoconstriction in EA. This shows their potential relevance in biomarker research (Watson et al. 1992). More recently, lipid profiling has expanded beyond particular eicosanoids toward broader FA signatures and extracellular vesicle (EV) cargo. Höglund et al. (2023) showed that FA profiles in BALF, BALF supernatant, and bronchoalveolar EVs differ between controls and horses with mEA and sEA. Across sample types, dihomo‑γ‑linolenic acid (20:3n-6) was consistently increased in asthma. EVs from severe cases showed decreased palmitic acid (16:0) and increased eicosapentaenoic acid (EPA; 20:5n-3). These data support a role of EV lipid cargo in asthma pathogenesis and resolution. Adding to the current findings, surfactant lipidomics derived from BALF presented a significant reduction in total phospholipid content in sEA and phenotype-dependent alterations. sEA was characterised by decreased phosphatidylglycerol (PG) and increased cyclic phosphatidic acid (cPA). Neutrophilic mEA showed reductions in selected phosphatidylglycerol (PG) species, accompanied by increased cPA. No significant surfactant lipid changes were detected in eosinophilic mEA. These results support airway lipid homeostasis as a severity-associated biomarker domain (Christmann et al. 2022).

### Neuromediators

Neuromediators are investigated as markers and therapeutic targets. NKA, a tachykinin family member, is recognised for strong pro-inflammatory effects. NKA binds preferentially to NK2 receptors (NKA > NKB > SP), with lower affinity for NK1 and NK3 receptors (Nederpelt et al. 2016; Pavón-Romero et al. 2021). NK2 receptor activation triggers airway smooth muscle contraction. Abnormal NK2 expression correlates with various forms of asthma in humans (Schelfhout et al. 2009; Morini et al. 2021; Pavón-Romero et al. 2021). In horses, NKA immunoreactivity increases after corticosteroid treatment, defying expectations. This demonstrates that clinical improvement does not necessarily resolve all molecular mechanisms of inflammation. The paradox hints at unique NKA regulation, supporting direct NK2 receptor targeting rather than reliance on corticosteroids (Morini et al. 2021).

### Proteomic profiling

Proteomic analysis of BALF is a more advanced approach for characterising BALF than cytology or single-analyte measurements. It enables large-scale protein detection with mass spectrometry (LC-MS/MS). Feutz et al. (2012) used this method to compare BALF from healthy horses and horses with sEA. Their analysis showed differences in peptide profiles between groups, but only some could be confidently matched to parent proteins (Feutz et al. 2012). They further validated two proteins, SCGB and TF. Contrary to earlier reports of reduced SCGB expression in sEA (Katavolos et al. 2009), Feutz et al. (2012) found no significant differences in SCGB expression between sEA and control. The authors attributed this to mild pulmonary inflammation during the exposure trial, subject variability, and limited statistical power. In contrast, TF expression tended to be lower in affected horses, and Western blot confirmed a significant reduction after allergen exposure, though inter-individual variation was large. The study’s limitations included a small sample size, use of a single hay-exposure model, and analysis limited to BALF supernatant. Despite these limitations, the results demonstrate the potential of BALF proteomics to discover respiratory biomarkers and suggest that molecular profiling may help us understand the mechanisms underlying sEA. The role of these proteins was more clearly defined in a subsequent study by Miskovic Feutz et al. (2015). It showed that SCGB and TF expression were decreased in horses with sEA. This was seen in both experimentally induced and naturally occurring disease, compared with healthy controls. The reduction was more pronounced for SCGB. This protein was significantly lower in sEA horses than in those with mEA. This suggests a potential role for SCGB for differentiating between EA phenotypes. Both proteins showed negative correlations with clinical score, airway obstruction, and BALF neutrophilia. In contrast, TF expression correlated positively with mast cell percentages. SCGB is an anti-inflammatory protein secreted by airway epithelial cells. It appears promising as a biomarker due to the consistent reduction in sEA and its pathophysiologic relevance. TF, while also decreased, may indicate wider inflammatory changes. It may provide less specificity as a diagnostic marker.

Beyond identifying individual biomarkers such as SCGB and TF, recent BALF proteomic studies have uncovered broader pathway-level signatures in sEA. In pasture-associated sEA, cell-free BALF proteomics showed a coordinated protein pattern reflecting increased neutrophil recruitment and tissue infiltration, with predicted suppression of neutrophil phagocytosis, respiratory burst, and apoptosis. These results indicate that proteomic signatures may capture the functional neutrophilic endotype more effectively than single-analyte biomarkers (Bright et al. 2019).

Mainguy-Seers et al. (2022) identified two additional proteins as potential sEA biomarkers. Specifically, proteomic profiling of neutrophil-derived EV highlighted LRCH4 and MAST4. Importantly, both proteins were increased during clinical exacerbation compared with remission, suggesting they may reflect neutrophil activation in neutrophil-predominant sEA. However, this association remains to be validated in larger, independent groups.

### Substances considered unsuitable as biomarkers

Despite a strong pathophysiological rationale, several candidate biomarkers have failed to show clinically meaningful differences between EA-affected and healthy horses. This reveals the difference between a hypothetical promise and clinical utility. MSTN is one such example. In human medicine, MSTN has been linked to pulmonary disease, such as COPD and asthma. Its overexpression can promote apoptosis in airway smooth muscle cells (Tan et al. 2022; Akenroye et al. 2023). This gives a theoretical rationale for its evaluation as a biomarker in EA. Kowalik et al. (2024) conducted the first systematic study of MSTN in this context. They found no significant differences in plasma MSTN concentrations between EA-affected and healthy adult leisure horses. This suggests MSTN is not a reliable diagnostic marker for EA. Instead, plasma MSTN levels in horses seem more influenced by breed, age, and physical activity than by asthma status. This case also points out the need to consider interspecies differences when comparing data across studies, as findings from human respiratory disease may not directly apply to EA (Kowalik et al. 2024).

PCT is another example of a biomarker with limited diagnostic value in EA. One study assessed its usefulness in horses with chronic pulmonary diseases, specifically sEA, mEA and CIP. The authors concluded that plasma PCT concentrations may assist in the overall assessment of chronic pulmonary disease in horses, but do not allow differentiation between specific disease entities. Moreover, PCT measured in BALF did not provide superior diagnostic accuracy compared with currently used clinical markers (Barton et al. 2016a).

In humans, vitamin D deficiency has been linked to asthma development, increased symptom severity, poorer disease control, reduced lung function, and a suboptimal response to corticosteroid therapy (Sutherland et al. 2010; Nanzer et al. 2013; Andújar-Espinosa et al. 2021). A retrospective study assessing serum 25(OH)D concentrations in healthy horses and in those with mEA and sEA found no significant differences between groups (Mainguy-Seers et al. 2024).

CICs have also been investigated as a possible systemic biomarker in sEA. In an environmental challenge model including healthy and asthmatic horses with and without corticosteroid treatment, CIC absorbance did not differ significantly between groups at any single time point, indicating limited diagnostic utility. However, longitudinal analysis showed CICs decreased in treated and increased in untreated asthmatic horses by day 30, suggesting their value for monitoring treatment response rather than diagnosing EA (Slowikowska et al. 2021).

Mason et al. (2018) demonstrate that extensive regulatory genetic variation, including cis- and trans-expression quantitative trait loci (eQTLS), can shape gene expression in peripheral blood mononuclear cells. This process occurs in a stimulus-dependent manner and helps explain why blood transcriptomic biomarker signals in sEA can vary across studies and conditions. Genome-wide association study signals for sEA overlapped with expression quantitative trait loci regulating DEXI. However, DEXI expression was not significantly associated with disease status. Additionally, none of the top 15 recurrent airway obstruction–associated loci appeared to affect disease by regulating gene expression in these blood cells.

### Candidate biomarkers in alternative biological matrices

Bazzano et al. (2020) applied 1 H-NMR metabolomics to BALF and EBC from horses with sEA and healthy controls. In their analysis of BALF, twelve metabolites were identified, with significant changes observed in four: decreased myo-inositol and glycerol, and increased formate and isopropanol in sEA. Similarly, in EBC, seven metabolites were detected; asthmatic horses exhibited higher methanol and ethanol levels and lower lactate levels. Taken together, this evidence shows distinct metabolic signatures in asthmatic horses and suggests myo-inositol (in BALF) and methanol (in EBC) as possible biomarkers. Furthermore, the study emphasises the promise of EBC as a noninvasive diagnostic matrix, though methodological standardisation is still lacking. In another study, EBC pH and H₂O₂ were increased in horses with lower airway inflammation and were positively associated with BALF neutrophilia, supporting their potential as non-invasive markers of neutrophilic airway inflammation. EBC LTB₄ was positively associated with BALF eosinophil percentage, suggesting a possible association with eosinophilic inflammation, but environmental and methodological factors substantially affected all analytes (Du Preez et al. 2019).

Lee et al. (2022) evaluated BALF, bronchial brush cytology, endobronchial biopsy, and salivary scavenger and agglutinin (SALSA) immunohistochemistry in sEA. While BALF and brush cytology detected marked post-challenge neutrophilia, SALSA immunolabelling intensity did not differ between asthmatic and control horses.

## Conclusion

EA is a prevalent and performance-limiting disease. Current diagnostic standards still rely on relatively invasive lower-airway sampling. The growing number of studies in this field highlights the importance of biomarker research. Available evidence suggests that both blood- and BALF-derived biomarkers may improve the diagnosis, phenotyping, and monitoring of EA. However, most candidates remain at an exploratory stage. No single biomarker has yet been identified that can reliably diagnose EA or consistently distinguish between its phenotypes. In blood, the most relevant candidates are SP-D, haptoglobin, and SCGB, with the best results obtained when these markers are interpreted in combination rather than individually. In BALF, the most informative candidates currently include NET-related markers, NGAL and allergen-specific IgE profiles. Current evidence increasingly suggests that biomarker research should be stratified according to the type of airway inflammatory infiltrate and the phenotype. Different forms of EA are likely to involve distinct immunological pathways. At the same time, the literature continues to be constrained by small study populations, heterogeneous case definitions, incompletely characterised control groups, and non-standardised sampling and analytical protocols. These issues limit translation into routine clinical practice. Future studies should therefore focus on larger study groups and harmonised methodologies. Longitudinal designs that allow assessment of biomarker changes over time in relation to disease progression, treatment response, and remission are also important. Importantly, the current data suggest that the most reliable diagnostic results are achieved with multimarker panels rather than single analytes assessed in isolation. Even so, biomarkers are more likely to complement existing diagnostic approaches than to provide a definitive diagnosis on their own. Such efforts will be essential for formulating robust, minimally invasive biomarker panels that can support, rather than replace, current diagnostic approaches in EA.

## Data Availability

Not applicable (no new data were generated).

## Abbreviations

8–OHdG: 8–hydroxy–2–deoxyguanosine
25(OH)D: 25–hydroxyvitamin D
6–keto–PGF1α: 6–keto–prostaglandin F1 alpha
ACVIM: American College of Veterinary Internal Medicine
ANCAs: anti–neutrophil cytoplasmic antibodies
AOPP: advanced oxidation protein products
APPs: acute–phase proteins
BALF: bronchoalveolar lavage fluid
cPA: cyclic phosphatidic acid
CICs: circulating immune complexes
CIP: chronic interstitial pneumopathy
COPD: chronic obstructive pulmonary disease
CRP: C–reactive protein
CXCL: C–X–C motif chemokine ligand
DEXI: dexamethasone–induced protein
EA: equine asthma
EBC: exhaled breath condensate
EMA: European Medicines Agency
EMMPRIN: extracellular matrix metalloproteinase inducer
EPA: eicosapentaenoic acid
ET: 1–endothelin–1
eQTLs: expression quantitative trait loci
EV: extracellular vesicle
FA: fatty acid
FeNO: fractional exhaled nitric oxide
FRAP: ferric reducing antioxidant power
GSHred: glutathione reductase
HOARSI: Horse Owner Assessed Respiratory Signs Index
HA: hyaluronic acid
IAD: inflammatory airway disease
IFN: γ–interferon gamma
KIT: KIT proto–oncogene, receptor tyrosine kinase
LC: MS/MS–liquid chromatography–tandem mass spectrometry
LRCH4: leucine–rich repeats and calponin–homology domain–containing 4
LTB₄: leukotriene B4
LTC₄: leukotriene C4
MANS: myristoylated N–terminal sequence
MARCKS: myristoylated alanine–rich C kinase substrate
MAST4: microtubule–associated serine/threonine–protein kinase 4
mEA: mild–to–moderate equine asthma
miRNAs: microRNAs
MMP: matrix metalloproteinases
MPO: myeloperoxidase
MSTN: myostatin
NETs: neutrophil extracellular traps
NGAL: neutrophil gelatinase–associated lipocalin
NGS: next–generation sequencing
NKA: neurokinin A
NKB: neurokinin B
PBS: phosphate–buffered saline
PCT: procalcitonin
PG: phosphatidylglycerol
PGD₂: prostaglandin D2
PGE₂: prostaglandin E2
PGF₂α: prostaglandin F2 alpha
PGI₂: prostacyclin
PIP3: phosphatidylinositol (3,4,5)–trisphosphate
RAO: recurrent airway obstruction
ROS: reactive oxygen species
SAA: serum amyloid A
SALSA: salivary scavenger and agglutinin
SCGB: secretoglobin
scRNA: seq–single–cell RNA sequencing
sEA: severe equine asthma
SOD: superoxide dismutase
SP: D–surfactant protein D
sTREM: 1–triggering receptor expressed on myeloid cells–1
TEAC: Trolox equivalent antioxidant capacity
TIMP: tissue inhibitors of metalloproteinases
TMS: tracheal mucus score
TNF: α–tumor necrosis factor alpha
TF: transferrin
TW: tracheal wash
TXB₂: thromboxane B2
